# Loss of Arabidopsis *GAUT12/IRX8* causes anther indehiscence and leads to reduced G lignin associated with altered matrix polysaccharide deposition

**DOI:** 10.3389/fpls.2014.00357

**Published:** 2014-07-28

**Authors:** Zhangying Hao, Utku Avci, Li Tan, Xiang Zhu, John Glushka, Sivakumar Pattathil, Stefan Eberhard, Tipton Sholes, Grace E. Rothstein, Wolfgang Lukowitz, Ron Orlando, Michael G. Hahn, Debra Mohnen

**Affiliations:** ^1^Department of Plant Biology, University of GeorgiaAthens, GA, USA; ^2^Complex Carbohydrate Research Center, University of GeorgiaAthens, GA, USA; ^3^BioEnergy Science Center (BESC), Oak Ridge National LaboratoryOak Ridge, TN, USA; ^4^Department of Chemistry, University of GeorgiaAthens, GA, USA; ^5^Department of Biology, Lawrence UniversityAppleton, WI, USA; ^6^Department of Biochemistry and Molecular Biology, University of GeorgiaAthens, GA, USA

**Keywords:** secondary cell walls, xylan, lignin, pectin, wall glycan epitopes, anther dehiscence

## Abstract

GAlactUronosylTransferase12 (GAUT12)/IRregular Xylem8 (IRX8) is a putative glycosyltransferase involved in Arabidopsis secondary cell wall biosynthesis. Previous work showed that Arabidopsis *irregular xylem8* (*irx8*) mutants have collapsed xylem due to a reduction in xylan and a lesser reduction in a subfraction of homogalacturonan (HG). We now show that male sterility in the *irx8* mutant is due to indehiscent anthers caused by reduced deposition of xylan and lignin in the endothecium cell layer. The reduced lignin content was demonstrated by histochemical lignin staining and pyrolysis Molecular Beam Mass Spectrometry (pyMBMS) and is associated with reduced lignin biosynthesis in *irx8* stems. Examination of sequential chemical extracts of stem walls using 2D ^13^C-^1^H Heteronuclear Single-Quantum Correlation (HSQC) NMR spectroscopy and antibody-based glycome profiling revealed a reduction in G lignin in the 1 M KOH extract and a concomitant loss of xylan, arabinogalactan and pectin epitopes in the ammonium oxalate, sodium carbonate, and 1 M KOH extracts from the *irx8* walls compared with wild-type walls. Immunolabeling of stem sections using the monoclonal antibody CCRC-M138 reactive against an unsubstituted xylopentaose epitope revealed a bi-lamellate pattern in wild-type fiber cells and a collapsed bi-layer in *irx8* cells, suggesting that at least in fiber cells, GAUT12 participates in the synthesis of a specific layer or type of xylan or helps to provide an architecture framework required for the native xylan deposition pattern. The results support the hypothesis that GAUT12 functions in the synthesis of a structure required for xylan and lignin deposition during secondary cell wall formation.

## Introduction

The plant extracellular matrix (i.e., cell wall) consists of a variety of complex carbohydrate polymers with distinct chemical and physical properties. The covalent and noncovalent interactions between these polymers in the final composite determine many of the characteristics of the cell wall. Accordingly, mutations in individual glycosyltransferases (GTs), each of which presumably participates in the biogenesis of a single cell wall component or domain, often affect multiple classes of cell wall polymers and sometimes result in dwarf plants. For example, the Arabidopsis mutant *irx7* (Zhong et al., 2005) is defective in both xylan and cellulose deposition, whereas *qua1* (Bouton et al., 2002; Leboeuf et al., 2005; Orfila et al., 2005), *parvus-3/gatl1* (Lao et al., 2003; Shao et al., 2004; Brown et al., 2007; Lee et al., 2007b; Kong et al., 2009), and *irx8*/*gaut12* mutants (Peña et al., 2007; Persson et al., 2007) are affected in pectin and xylan biosynthesis. These complex effects make it difficult to infer primary gene function on the basis of mutant phenotypes alone.

The gene mutated in the xylan- and pectin-deficient mutant *irregular xylem8* (*irx8*) is *GAUT12/IRX8* (*At5g54690*), a member of CAZy family GT8 that contains GTs related to homogalacturonan (HG):α-1,4-galacturonosyltransferase (GalAT) GAUT1 (Sterling et al., 2006). In this paper we refer to the gene affected in the *irx8* mutant as *GAUT12* and its protein as GAlactUronosylTransferase12 (GAUT12). GAUT12 is predicted to be a type II transmembrane protein with its C-terminal catalytic domain facing the Golgi lumen. Transient expression of YFP-tagged GAUT12 protein showed that it co-localizes with CFP-tagged MUR4, consistent with the localization of GAUT12 in the Golgi apparatus (Peña et al., 2007). Transcription of *GAUT12* is strongest in xylem vessels and interfascicular fiber cells, and *irx8* mutant cell walls show a substantial reduction in glucuronoxylan (Peña et al., 2007; Persson et al., 2007) as well as a modest reduction in α-1,4-linked GalA (Persson et al., 2007). Xylan is one of the major components of the secondary wall, and pectin is a major matrix polysaccharide in primary walls, but is also found in low abundance in walls prepared from cells synthesizing secondary walls. Additionally, *irx8* mutant xylan is nearly devoid of a xylan reducing-end glycosyl sequence [XRES; β-d-Xyl*p*-(1→3)-α-l-Rha*p*-(1→2)-α-d-Gal*p*A-(1→4)-d-Xyl*p*] (Peña et al., 2007). XRES has been proposed to act either as a primer to initiate xylan biosynthesis or a terminator to control xylan chain length (York and O'Neill, 2008). However, it remains unknown how XRES synthesis is initiated. Since stem microsomes isolated from *irx8* plants contain comparable amounts of xylan:xylosyltransferase and xylan:glucuronosyltransferase activity as their wild-type counterparts (Brown et al., 2007; Lee et al., 2007a), it seems unlikely that GAUT12 is involved in the elongation or branching of the xylan backbone (York and O'Neill, 2008; Scheller and Ulvskov, 2010). Based on analyses of *irx8* cell walls and GAUT12 protein homology to GAUT1, it has been hypothesized that GAUT12 is a GalAT that either synthesizes a subfraction of HG (Persson et al., 2007) or catalyzes the addition of GalA into the nascent XRES (Peña et al., 2007). The biochemical function of GAUT12, however, remains unresolved to date.

In addition to being severely dwarfed and slow growing, Arabidopsis *irx8* mutants are sterile (Persson et al., 2007). Consistent with a role in secondary wall formation and reproduction, *GAUT12* expression is regulated by transcription factors that regulate vessel and fiber formation, such as MYB46 (Ko et al., 2009), MYB83 (McCarthy et al., 2009), VND6, and VND7 (Yamaguchi et al., 2010), as well as by transcription factors that act in anther development, such as MYB26/MALE STERILE35 (Steiner-Lange et al., 2003; Yang et al., 2007), NST1/NST2 (Mitsuda et al., 2005), and AHP4 (Jung et al., 2008). Within anthers, secondary wall thickenings in the endothelium cell layer provide part of the biophysical force that enables dehiscence, the programmed rupture of the anthers to release mature pollen (Wilson et al., 2011). Several lignin-defective mutants have recently been shown to be indehiscent and to generate defective pollen grains (Schilmiller et al., 2009; Weng et al., 2010; Thevenin et al., 2011).

The phenotypes of the Arabidopsis *irx8* mutant include not only a loss of ~60% xylan and ~13% pectin, but also ~25% cellulose. This is a significantly smaller reduction in cellulose than observed in the cellulose defective mutants *irx1* and *irx3* (Brown et al., 2005; Persson et al., 2005). Based on phloroglucinol-HCl staining, a reduction of lignin in *irx8* vessels and fiber cells was also recently reported that was suggested to be associated with reduced xylan biosynthesis (Petersen et al., 2012). Lignin, a resin-like molecular network generated by oxidative polymerization of phenolic subunits within the extracellular space of many terminally differentiated cells constitutes up to 30% of most secondary cell walls (Boerjan et al., 2003). The three subunits of lignin, namely *p*-hydroxyphenyl (H), guaiacyl (G), and syringyl (S) are laid down in specific spatio-temporal patterns. For example, in wood formation H and G lignins are deposited at early stages of lignification in the middle lamella and tricellular junctions, while G lignin is deposited earlier than S lignin in vessels and fibers and S lignin is mainly deposited in fibers (Donaldson, 2001). Lignin subunits also become covalently linked to hemicelluloses and pectins (Jeffries, 1990), and the carbohydrate polymers are thought to guide expansion of the lignin lamellae during lignification (Donaldson, 2001; Donaldson and Knox, 2012).

The goal of this study was to elucidate the biological function(s) of *GAUT12* in Arabidopsis. We identified the cause of sterility in the *irx8* mutant and a reduction in lignin in this mutant. We attempted to solve the biochemical function of GAUT12 and found that GAUT12 does not have HG:GalAT activity comparable to that of GAUT1. In addition, an increased expression of an RG-I epitope in *irx8* fiber cell walls was revealed. Our results suggest a connection between an RG-I-containing structure and the *GAUT12*-dependent wall product. We propose that GAUT12 participates in the synthesis of a structure required for xylan and lignin deposition during the formation of the secondary cell wall and that pectin is associated with this structure.

## Materials and methods

### Plant materials

Arabidopsis wild type (*Col-0*), *irx8-5* (SALK_044387), *irx8-2* (SAIL_603_G02), *parvus-3* (SALK_045368), and *irx9-1* (SALK_058238) plants were grown on soil in a controlled-environment chamber (Conviron, Pembina, ND) under a 14-h-light/10-h-dark cycle at 19 and 15°C, respectively. Light intensity was 150 μEm^−2^s^−1^ and relative humidity was maintained at 50%. Plants were harvested after 7–8 weeks. T-DNA insertions were confirmed using primers from genomic regions flanking the T-DNA and the general T-DNA left border primer (Supplemental Table S1). Arabidopsis *Col-0* and *irx8* heterozygote plants were transformed via the floral dip method (Clough and Bent, 1998) and transgenic plants selected on ½ MS media plates containing 15 mg/L hygromycin. Transgenic plants harboring the construct in *Col-0* and *irx8* homozygote mutant backgrounds were genotyped using PCR (Primers listed in Supplemental Table S1).

### Generation of the *GAUT12-EGFP* construct

*GAUT12* coding sequence (CDS) was amplified from total RNA (0.5 μg) isolated from 7-week-old Arabidopsis *Col-0* stem by RT-PCR using the SuperScriptTM III One-Step RT-PCR System with Platinum Tag High Fidelity (Invitrogen 12574-030) and cloned into pGEM®-T Easy vector (Promega) (primers listed in Supplemental Table S1). The amplified *GAUT12* CDS was sequence-verified and cloned into the over-expression construct pCambia35tl:egfps2#4 (Pattathil et al., 2005) between the NcoI and KpnI restriction sites to produce the *GAUT12-EGFP* construct driven by the CaMV 35S promoter. The plasmid was electroporated into *Agrobacterium tumefaciens* strain GV3101 competent cells and the transformed cells used to transform both wild-type (WT) and heterozygous *irx8* Arabidopsis plants.

### Histochemical staining

Mäule reagent was prepared as described (Chapple et al., 1992) with slight modifications and used to detect S lignin. Arabidopsis open flowers and hand-cut stem transverse sections were treated with 0.5% (w/v) KMnO_4_ solution for 10 min and rinsed with water. For flower samples, the solution was supplemented with 0.01% (v/v) 7X detergent (Linbro, Flow Laboratories) to break surface tension. Samples were treated with 10% (v/v) HCl for 5 min, rinsed with water and mounted in concentrated ammonia for microscopic observation.

Phloroglucinol-HCl stain was prepared freshly as described (Guo et al., 2001). Two parts of 2% (w/v) phloroglucinol in 95% (v/v) ethanol were mixed with one part concentrated HCl. Pictures were taken 10 min after applying the stain. Stained flowers were viewed using a dissecting scope (Olympus SZH) under dark field. Stained stem transverse sections were viewed using a Nikon Eclipse80i microscope under bright field. Images were captured using a Nikon DS-Ril camera head (Nikon, Melville, NY).

### Scanning electron microscopy

Using a dissecting scope, anthers from WT and mutant open flowers were removed with dissecting forceps (Sigma-Aldrich T4537). Pollen was released onto specimen stubs topped with double-sided sticky carbon tabs by gently tapping the forceps, or lightly tapping the anthers onto the stubs. The *irx8* mutant anthers were first manually dissected to open them and pollen was gently scooped out using the forceps tip and transferred onto the stub surface. Samples were dehydrated and coated with gold particles for 120 s in a Sputter Coater, and imaged using either a JEOL JSM-5800 (SEM/EDAX) scanning electron microscope or a Topcon Aquila—Hybrid SEM.

### *In vitro* pollen tube growth and RNA preparation

Pollen tubes were grown *in vitro* as previously described (Dardelle et al., 2010). In brief, WT pollen grains were collected from 40 open flowers by vortexing for 3 min in a microcentrifuge tube containing 1 ml pollen germination medium (PGM) composed of 5 mM CaCl_2_·2H_2_O, 0.01% (w/v) H_3_BO_3_, 5 mM KCl, 1 mM MgSO_4_·7H_2_O, and 10% (w/v) sucrose (pH adjusted to 7.5 using KOH). The flowers were carefully removed and pollen grains pelleted by 3200 *g* centrifugation for 6 min. The old media was removed, the pollen pellet gently re-suspended in 250 μL of fresh (PGM), and the pollen grains transferred into a 13 × 100 mm glass tube, covered with 3M micropore tape and set in the dark at 22°C for 6 or 24 h.

For RNA isolation, pollen grains from 200 open WT flowers were collected in PGM in five tubes. Pollen from 200 flowers was either directly harvested as hydrated pollen grains (0.5 h) or grown as pollen tubes for 6 and 24 h. Hydrated pollen grains (0.5 h) were combined and ground in liquid nitrogen using a plastic pestle and a microcentrifuge tube. Pollen tubes grown for 6 h and 24 h for RNA isolation were collected by centrifugation for 6 min at 3200 *g* and ground in microcentrifuge tubes. RNA isolation was repeated using three batches of independently collected tissues.

### Tissue fixation and immunolabeling

Freshly cut plant tissues were fixed in 25 mM sodium phosphate buffer (pH 7.1) with 1.6% (w/v) paraformaldehyde and 0.2% (w/v) glutaraldehyde overnight at 4°C. Using a lab-grade microwave (PELCO BioWave Pro, Ted Pella, CA) set at 250 Watt, tissues were washed three times for 1 min each with 25 mM sodium phosphate buffer (pH 7.1) followed by two washes with water. Samples then underwent a series of 40-s ethanol gradient incubations (35, 50, 75, 95, 100, 100, and 100% [v/v]) to dehydrate the tissue. Samples were infiltrated with cold LR White embedding resin (Ted Pella) in a gradient (1:3, 1:1, 3:1 resin:ethanol [v/v]) and finally three times with 100% resin. Each step was conducted under vacuum (20″ Hg) for 2.5 min. After the last resin change, samples were kept at 4°C for 24 h, transferred into gelatin capsules filled with resin, and polymerized under 365 nm UV light at 4°C for 48 h. Tissue cross sections (250 nm) were cut with a Leica EM UC6 ultramicrotome (Leica Microsystems), mounted on pre-coated slides (Colorfrost/Plus, Fisher Scientific) and used for immunolabeling or stained with 0.05% (w/v) toluidine blue for light microscopy.

Immunolabeling for fluorescent microscopy was done as described (Avci et al., 2012). For LM series and JIM series antibodies, a wash buffer containing 10 mM KPBS (pH 7.2) and 100 mM NaCl was used because we found that the use of 500 mM NaCl adversely affected consistent binding of these antibodies. The secondary antibody used for the LM and JIM series was Alexa fluor 488 goat anti-rat IgG (Cat#A11006, Invitrogen) which was applied to the sections in the same manner as described above, but diluted in the low salt wash buffer. Images were captured using a Nikon DS-Ril camera head (Nikon, Melville, NY). All data shown depict representative images out of three images viewed for each type of tissue section stained with each antibody.

### Transmission electron microscopy

Ultrathin sections (80 nm) were prepared using an ultramicrotome (Leica EM UC6, Austria) and collected on Formvar-coated nickel grids. Grids were stained with 2% (w/v) uranyl acetate for 4 min followed by 10 dips in three changes of deionized water and dried by wicking. Micrographs were recorded on film in a JEOL 100S transmission electron microscope. The negatives were developed and scanned in Adobe Photoshop.

For immunogold labeling, ultrathin sections were blocked in TBS (10 mM Tris buffer, 150 mM NaCl, pH 7.5) containing 0.06% (w/v) bovine serum albumin for 30 min at room temperature in a petri-dish with a folded piece of water-soaked Kimwipe set to one side of the dish. Sections were transferred onto 10 μL drops of primary antibody diluted (1:5) in TBS for 1 h. After washing three times by dipping the grids (10 times) in TBS, secondary antibody (goat anti-rat IgG coupled to 15 nm gold,) diluted 1:10 in TB (10 mM Tris buffer) containing 0.06% (w/v) bovine serum albumin was applied to the sections. The sections were dipped in TB and distilled water for washing and dried by wicking. Images shown are representative of four images viewed for each sample stained with each antibody.

### Stem RNA isolation and quantitative PCR analyses

The bottom half of stem tissues from wild type and *irx8*, in which secondary cell walls are actively synthesized, were flash frozen in liquid nitrogen and ground to a fine powder in liquid nitrogen in pre-chilled mortars with pestles. Total RNA was isolated from ~100 mg of frozen powder from three individual tissue samples using RNeasy Plant Mini Kit (Qiagen, 74904). First-strand cDNA was synthesized from 1 μg of total RNA using SuperSript III First-Strand Synthesis Super mix (Invitrogen, 18080-400) followed by quantitative PCR analysis using iQ™ SYBR Green Supermix (Bio-Rad 170-8882) on a CFX96™ Real-Time PCR Detection System (Bio-Rad) following the manufacturer's instructions. Melt curve analyses were performed after each run to ensure single size amplicon production. Data are the average ± standard deviation of three biological samples. The data were analyzed as described (Livak and Schmittgen, 2001). Primer sequences are provided in Supplemental Table S1.

### Cell wall (AIR) preparation

Whole stem tissues were harvested from 7-week-old *Col-0*, *irx8-5*, *irx8-5+GAUT12*, *irx8-2+GAUT12*, and WT+*GAUT12*, ground with a mortar and pestle to a fine powder in liquid nitrogen, re-suspended in 80% (v/v) ethanol and rotated end-to-end for 12 h. The pellet obtained upon 4000 rpm centrifugation was sequentially washed in 80% (v/v) ethanol, 100% ethanol, chloroform: methanol (1:1, v/v), and acetone by re-suspension, rotation for 12 h, and re-centrifugation. The resulting alcohol insoluble residue (AIR) was dried for 24 h at room temperature.

### Glycosyl residue composition analyses

Neutral sugar analysis was performed as described (Albersheim et al., 1967) with slight modifications. Each AIR sample (0.4 mg with 20 μg *myo*-inositol as an internal standard) was hydrolyzed with 30 drops of 2 N trifluoroacetic acid (TFA) for 2 h at 120°C. Samples were cooled to room temperature and dried under an air stream, washed twice with isopropanol to remove TFA, and reduced by incubation for 1 h at room temperature in 10 drops of sodium borohydride (10 mg/ml) dissolved in 1 M ammonium hydroxide solution. The reaction was quenched with 30–40 drops of acetone and dried down with air. Once the volume was reduced to half, isopropanol (1 ml) was added to each sample to facilitate drying. *O*-acetylation was performed by adding 250 μl of acetic anhydride followed by 230 μl of concentrated TFA and the samples were incubated for 10 min at 50°C. Samples were then washed with isopropanol and dried down with air. H_2_O (1 ml) and dichloromethane (DCM, 1 ml) were added and the samples vortexed and centrifuged to allow phase separation. The top aqueous layer was discarded and the bottom DCM layer containing the alditol acetate derivatives was dried down. Ten drops of DCM were added to each sample before analysis by gas-liquid chromatography-flame ionization detection using an Agilent 7890A GC system.

For GalA and GlcA measurements, AIR samples (0.5 mg) were hydrolyzed in 2N TFA at 105°C for 1 h. The hydrolysates were dried down with an air stream, re-hydrolyzed in 3N methanolic HCl (Thermo Scientific, Rockford, IL) overnight at 80°C, dried down individually and dissolved in 100 μl distilled H_2_O. The samples were centrifuged and 12.5 μl of hydolysate supernatant was loaded onto a CarboPac PA20 analytical column (3 × 150 mm, Dionex, Sunnyvale, CA). The loaded column was washed with a solution of 49 mM NaOH, 20 mM NaOAc and eluted with a linear gradient from 49 mM NaOH, 20 mM NaOAc to 40 mM NaOH, 200 mM NaOAc over 25 min. The column was eluted at 30°C and a flow rate of 0.4 ml/min and effluent was monitored with an ECD detector. The amount of GalA and GlcA was determined by comparison of peak areas to standards separated under the same conditions.

Each analysis was repeated five times for each sample, and the bar represents the average mol% of each sugar residue ± standard error.

### Sequential extraction and glycome profiling

Sequential extractions of cell wall samples and glycome profiling were carried out as described previously (Demartini et al., 2011; Pattathil et al., 2012). Briefly, AIR samples were sequentially extracted with 50 mM ammonium oxalate, pH 5; 50 mM sodium carbonate [containing 0.5% (w/v) sodium borohydride], pH 10; 1 M KOH with 1% (w/v) sodium borohydride; 4 M KOH with 1% (w/v) sodium borohydride; 100 mM acidified sodium chlorite; and finally with 4 M KOH with 1% (w/v) sodium borohydride for the post-chlorite extraction. The wall extracts were used for NMR analyses (see below) and were screened by ELISA using plant glycan-directed monoclonal antibodies (CCRC, JIM, and MAC series) from Complex Carbohydrate Research Center stocks available through CarboSource Services (http://www.carbosource.net). Detailed description of each mAbs used in this study can be found in the Supporting Information (Supplemental Table S2) that includes links to a web database named Wall*MAb*DB (http://www.wallmabdb.net).

### Pyrolysis molecular beam mass spectrometry (pyMBMS)

About 4 mg of sample was weighed and transferred into 80-μl stainless steel sample cups of an auto sampler of a double shot pyrolyzer (PY-2020iD, Frontier Ltd.). The samples were pyrolyzed at 500°C and the residues analyzed using a custom built Super Sonic Molecular Beam Mass Spectrometer (Extrel Model MAX-1000).

Mass spectral data from m/z 30–450 were acquired on a Merlin Automation Data System version 3.3. Multivariate analysis was performed using Unscrambler software version 10.1 (CAMO). The intensities of the lignin peaks were summed and averaged to estimate the lignin content in the sample (Evans and Milne, 1987). Total lignin peaks corresponded to m/z 120, 124, 137, 138, 150, 152, 154, 164, 167, 168, 178, 180, 181, 182, 194, 208, and 210. The syringyl peaks corresponded to m/z 154, 167, 168, 182, 194, 208, and 210, the guaiacol peaks corresponded to m/z 124, 137, 138, 150, 164, and 178, and phenol peaks corresponded to m/z 120 and 122. Syringyl to Guaiacol (S/G) ratios were determined by summing syringyl peaks and dividing by the sum of guaiacol peaks. The lignin values thus generated and calculated were compared with the WT. Because the NIST (National Institute Standards and Technology) standard for Arabidopsis is not available, the lignin percentage was corrected using a standard of eastern cottonwood (*NIST 8492*) to obtain the lignin percentage value equivalent to the Klason lignin. This analysis was conducted at the Complex Carbohydrate Research Center Analytical Services, University of Georgia (http://www.ccrc.uga.edu/services/ccrcanalyticalservices/index.html).

### Determination of lignin monomer composition by HSQC NMR spectroscopy

Perdeuterated pyridinium molten salt (ionic liquid) was synthesized as described (Jiang et al., 2009). Approximately 2 mg of cell wall extracts were weighed and dissolved in 180 μl of the ionic liquid [DMSO-*d*_6_/pyridine-*d*_5_ (2:1, v/v)] at 65°C. Data were collected at 60°C on Agilent 600 MHz Direct Drive spectrometers equipped with either a 5 or 3 mm cold probe. A standard Agilent pulse program (“HSQCAD”) was used to acquire the ^13^C-^1^H heteronuclear correlated spectra. The proton dimension of 1200 complex data points covered 20 ppm centered at 6 ppm, and the carbon dimension of 48 or 64 complex points was centered at 92 ppm with a width of either 100 or 184 ppm, respectively. In the former case, some resonances in the alkyl or aromatic regions were folded along the F1 axis. Total data acquisition times ranged from 7 to 16 h, with the number of transients between 240 and 400 per *t*_1_ increment. Data were processed with NMRPipe (NIH) and visualized with NMRViewJ (One Moon Scientific) or with MNova (Mestrelab Research). Typically, squared cosine window functions were applied in both dimensions after zero filling and linear prediction in *t*_1_. Chemical shifts were referenced to DMSO at 2.50 ppm in proton and 39.51 ppm in carbon. Heteronuclear Single-Quantum Correlation (HSQC) cross-peak assignments were determined and referenced as described (Kim and Ralph, 2010).

### Generation of arabidopsis suspension cultures and *GAUT12-EGFP* transgenic cells

A wild-type Arabidopsis suspension culture was generated from seed callus as previously described (Doelling and Pikaard, 1993). Cell were subcultured every 10 days by transfer of 7 ml of packed cells into 100 ml fresh callus inducing medium (CIM). CIM contained 3.2 g/L Gamborg's B-5 basal medium with minimal organics (Sigma-Aldrich G5893 or PhytoTechnology Laboratories G398), 2 mg/L 2,4-D, 0.05 mg/L kinetin, and 20 g/L sucrose (pH 5.7). The GAUT12-EGFP construct was stably transformed into wild-type suspension culture cells via *Agrobacterium tumefaciens* strain GV3101. An aliquot of 1 ml packed cells from a four-day-old culture was co-cultured in 8 ml of fresh CIM with 100 μl of Agrobacterium cells harboring the *GAUT12-EGFP* vector re-suspended in CIM to an OD_600_ of 0.8. The Agrobacteria were previously seed-cultured overnight in 3 ml YEP medium supplemented with rifampicin 50 mg/L, kanamycin 50 mg/L, and gentamycin 50 mg/L and re-cultured in 25 ml of the above YEP medium. The co-culture was done in a 1-inch-deep petri-dish in the dark on a gyrotory shaker at 130 rpm for 24 h. Cells were removed from the co-cultivation medium and washed thrice with CIM and then with CIM supplemented with 500 mg/L cefotaxime in 50 ml falcon tubes. In each wash, the old medium was completely removed and 20 ml fresh medium introduced and vortexed for 30 s. The washed cells were plated onto CIM 0.6% (w/v) agar plates containing 300 mg/L cefotaxime and 15 mg/L hygromycin. Hygromycin-resistant transgenic calli emerged in 2–3 weeks and were transferred onto fresh media plates and grown for up to 4 weeks. After two to three transfer cycles, the calli were free of Agrobacteria and used to initiate suspension cultures. Surviving calli were genotyped, and RT-PCR was used to determine the expression level of the *GAUT12-EGFP* transcript. The *GAUT12-EGFP* transcript was highest on day 5 compared to day 2 and 8. Therefore, cells were harvested on day 6 for preparation of microsomes.

### Microsomal membrane preparation

Microsomal membranes used for enzyme activity assays were prepared at 4°C as described (Orfila et al., 2005) with modification. Arabidopsis stems (10 grams) were cut into small pieces, flash frozen in liquid nitrogen, and homogenized on ice in 20 ml of pre-chilled homogenization buffer containing 50 mM Hepes (pH 7.3), 0.4 M sucrose, 0.1 M sodium ascorbate, 0.25 mM MnCl_2_, 25 mM KCl, 1% (w/v) polyvinylpyrrolidone (PVPP), and EDTA-free protease inhibitor cocktail (Roche) until the tissues were pureed. The homogenate was filtered through three layers of miracloth and the filtrate centrifuged at 4°C for 30 min at 4000 *g* to remove cell debris and intact cells. The supernatant was ultra-centrifuged at 110,000 *g* for 1 h, yielding the microsome pellet which was re-suspended on ice in pre-chilled storage buffer (homogenization buffer without PVPP, 30 μl buffer/gram fresh weight) using a glass homogenizer. Total protein was measured using the Bradford assay (Bio-Rad Protein Assay 500-0006) with BSA as a standard. Aliquots of the microsomes were used directly or flash frozen in liquid nitrogen and stored at −80°C for later use in enzyme assays and immunoprecipitation experiments.

### Generation of anti-GAUT12 polyclonal antibody and western blotting

The anti-GAUT12 polyclonal antibody was generated against synthetic peptides corresponding to GAUT12 amino acid residues 101–114 (EQPLSEQELKGRSD) and antigen-purified over a column packed with the antigenic-peptide (service via New England Peptide, http://www.newenglandpeptide.com/). The purified anti-GAUT12 antibody did not cross-react with GAUT1 or GAUT7 (Supplemental Figure S11). Pre-immune serum did not contain anti-GAUT12 antibody. Anti-GAUT1 and anti-GAUT7 antibodies were generated previously (Sterling et al., 2006; Atmodjo et al., 2011). For western blotting, a dilution factor of 1:5000, 1:10000, 1:3000, and 1:2000 was applied for anti-GAUT12, anti-GAUT7, anti-GAUT1, and anti-GFP (abcam, ab6556), respectively. Either horseradish peroxidase (HRP)- or alkaline phosphate (AP)-conjugated goat anti-rabbit secondary antibody (Sigma) was used followed by a reaction with corresponding substrates to yield a blue or purple color precipitant on the target protein band.

### Immunoprecipitation of GAUT12 for HG:GALAT enzyme assays

The purified anti-GAUT12 antibody (1.14 mg/ml) was incubated with Dynabeads M-280 Sheep anti-Rabbit IgG (Invitrogen, 112-04D) in a ratio of 1:20 (v/v) for 2 h at 4°C on a tube rotator and the beads collected on a magnet stand and washed thrice with isotonic PBS (pH 7.4, 139 mM NaCl, 5.5 mM Na_2_HPO_4_, 1.2 mM NaH_2_PO_4_). The beads were then washed once with storage buffer (see microsome preparation section). Each reaction contained 30 μl anti-GAUT12-conjugated beads incubated with ~500 μg Triton X-100-treated microsomes at 4°C for 2 h on the tube rotator. The nonionic detergent Triton X-100 (TX-100) has been used to solubilize GalAT activity during GAUT1 and GAUT7 purification (Doong and Mohnen, 1998; Sterling et al., 2006; Atmodjo et al., 2011). A concentration of 4% (v/v) TX-100 was used to homogenize WT stem microsomes on ice and they were immediately diluted with storage buffer to a final detergent concentration of 0.5% (v/v) for incubation with anti-GAUT12-conjugated magnetic beads.

After a 2 h end-to-end incubation at 4°C, the beads were washed with pre-chilled storage buffer on ice thrice and the original 30 μl beads were re-suspended in 13 μl pre-chilled storage buffer for each enzyme reaction. Immunoabsorbed-GAUT1 was prepared by incubating Dynabeads M-280 Sheep anti-Rabbit IgG with anti-GAUT7 anti-serum in a ratio of 1:3 (v/v) in parallel as described (Atmodjo et al., 2011). Anti-GAUT7 antibody was previously shown to immunoabsorb the HG:GalAT activity-containing the GAUT1-GAUT7 core complex (Atmodjo et al., 2011).

### HG:GALAT enzyme activity assay

UDP-d-[^14^C]Gal*p*A (specific activity 180.3 mCi/mmol; 1 Ci = 37 GBq) was synthesized enzymatically from UDP-d-[^14^C]Glc*p*A using UDP-d-Glc*p*A 4-epimerase as described (Atmodjo et al., 2011). HG:GalAT activity was assayed in 30-μL reactions containing either enzyme (15 μl total microsomes, ~100 μg total protein) or 13 μl of immunoabsorbed beads, 50 mM Hepes (pH 7.3), 0.2 M sucrose, 0.05% (w/v) BSA, 25 mM KCl, 1.9 mM MnCl_2_, 1 mM HG oligosaccharides (referred to as oligogalacturonides, OGA) of a degree of polymerization (DP) of 7–23, and 6.9 μM UDP-[^14^C]Gal*p*A (specific activity 180.3 mCi/mmol; 1 Ci = 37 GBq). The reactions were incubated for 3 h at 29°C in a water-bath and terminated by the addition of 5 μl of 400 mM NaOH with vortexing. The entire reaction (~35 μl) was spotted onto 1-inch^2^ filter paper squares that had been pre-treated with cetylpyridinium chloride (CPC) as described (Sterling et al., 2005). The spotted filters were dried, washed 3× in 150 mM NaCl each for 15 min to remove access UDP-[^14^C]Gal*p*A, dried, and added to 4 ml ScintiVerse ™ BD cocktail for scintillation counting.

### Liquid chromatography-tandem mass spectrometry (LC-MS/MS)

A large-scale GAUT12 immunoprecipitation was conducted for LC-MS/MS analysis. An aliquot of 500 μl of the anti-GAUT12-conjugated magnetic beads was incubated at 4°C overnight on a tube rotator with WT Arabidopsis stem microsomes (5 mg total protein). The microsomes on ice were homogenized with 4% (v/v) TX-100 supplemented with 200 mM NaCl, 100 mM NaOAc, and 2 mM EDTA and immediately diluted with PBS to a final detergent concentration of 0.5% (v/v) for incubation with anti-GAUT12-conjugated magnetic beads. The beads were washed 5× with PBS (pH 7.4) and the recovered beads denatured in 3% (w/v) SDS and reduced in 25 mM DTT. The material bound to the beads was released by magnetic separation and separated by electrophoresis on a 10% SDS-PAGE gel. A gel piece corresponding to the size of GAUT12 (between 55 and 70 KDa protein marker) was cut out and in-gel trypsin-digested as described (Atmodjo et al., 2011). The peptide samples from the proteolytic digestions were analyzed on an Agilent 1100 capillary LC (Palo Alto, CA) interfaced directly to a LTQ linear ion trap mass spectrometer (Thermo Fisher, San Jose, CA). Mobile phases A and B were H_2_O-0.1% (v/v) formic acid and acetonitrile-0.1% (v/v) formic acid, respectively. Peptides were eluted from the C18 column into the mass spectrometer during an 80 min linear gradient from 5 to 55% (v/v) mobile phase B at a flow rate of 4 μl/min. The instrument was set to acquire MS/MS spectra on the nine most abundant precursor ions. Generated raw tandem mass spectra were converted into the mzXML format and then into peak lists using ReAdW software followed by mzMXL2Other software (Pedrioli et al., 2004). The peak lists were searched using Mascot 2.2 (Matrix Science, Boston, MA).

### Database searching and protein identification

A target database was created using the Arabidopsis annotated sequences obtained from the TAIR10_pep_20101214 protein database (ftp://ftp.arabidopsis.org/home/tair/Genes/TAIR10_genome_release/TAIR10_blastsets/TAIR10_pep_20101214_updated). A decoy database (decoy) was constructed by reversing the sequences in the normal database. Searches were performed against the normal and decoy databases using the following parameters: full tryptic enzymatic cleavage with two possible missed cleavages, peptide tolerance of 1000 ppm, fragment ion tolerance of 0.6 Da. Fixed modification was set as carbamidomethyl due to carboxyamidomethylation of cysteine residues (+57 Da) and variable modifications were chosen as oxidation of methionine residues (+16 Da) and deamidation of asparagine residues (+1 Da). Statistically significant proteins from both searches were determined at a ≤1% protein false discovery rate (FDR) using the ProValT algorithm, as implemented in ProteoIQ (BioInquire, LLC, Athens, GA) (Weatherly et al., 2005).

## Results

### The effects of *irx8* mutation on growth habit and secondary wall synthesis in xylem and fibers are complemented by a *GAUT12*-overexpression construct

All reported *irx8* mutant alleles are dwarf and show a collapsed xylem phenotype (Peña et al., 2007; Persson et al., 2007). We selected two of these *GAUT12* T-DNA insertion mutants, *irx8-2* and *irx8-5*, for this study (Supplemental Figure S1A). The T-DNA insertions in *irx8-2* and *irx8-5* are in the promoter region and fourth intron of *GAUT12*, respectively (Supplemental Figure S1A). Both alleles are associated with phenotypes similar to those previously described (Peña et al., 2007; Persson et al., 2007). Specifically, both mutants have a dwarfed growth habit (Supplemental Figure S1B), reduced secondary cell wall thickness in xylem and fiber cells, and collapsed xylem vessels (Supplemental Figures S1D,H). Semi-quantitative RT-PCR analyses revealed that *irx8-2* plants contain trace amounts of full-length *GAUT12* transcript, whereas *irx8-5* plants contain transcripts that are truncated at the insertion site (Supplemental Figure S1L). Since both *irx8-2* and *irx8-5* mutants express similar overall phenotypes, they were used interchangeably in the present study.

The *irx8* mutant phenotypes in both alleles are complemented (Supplemental Figures S1E,I,K) by the constitutive expression of an EGFP-tagged *GAUT12* construct (*GAUT12-EGFP*). The EGFP (Pattathil et al., 2005) was connected to the C-terminus of GAUT12 via a Val-Pro linker to facilitate structural flexibility between the two parts of the fusion protein and access of substrates to the predicted C-terminal catalytic domain of GAUT12 (Supplemental Figure S1A). The *GAUT12-EGFP* construct restored the phenotype of *irx8* mutants as evidenced by the normal stature (Supplemental Figure S2B) and cell wall thickness (Supplemental Figures S3C,G) in the complemented transgenic plants. Cell wall sugar composition analyses revealed that *irx8-5*+*GAUT12* and *irx8-2*+*GAUT12* plants had a xylose content that was closer to normal (65 and 74 mol% of wild-type level, respectively) compared to the *irx8-5* mutant (33 mol% of wild-type xylose content, Supplemental Figure S2C). Finally, the 20 and 63 mol% reduction of GalA and GlcA content, respectively, associated with the *irx8-5* mutation was also complemented by the *GAUT12-EGFP* construct (Supplemental Figure S2D).

### Reduction in lignin and xylan leads to indehiscent anthers in *irx8*

Prior studies reported that multiple alleles of homozygous *irx8* mutants were “semi-sterile” (Brown et al., 2005; Persson et al., 2007). Both the *irx8-2* and *irx8-5* mutants used for this study produced small and empty siliques with almost no seeds (Supplemental Figure S1M).

**Figure 1:**
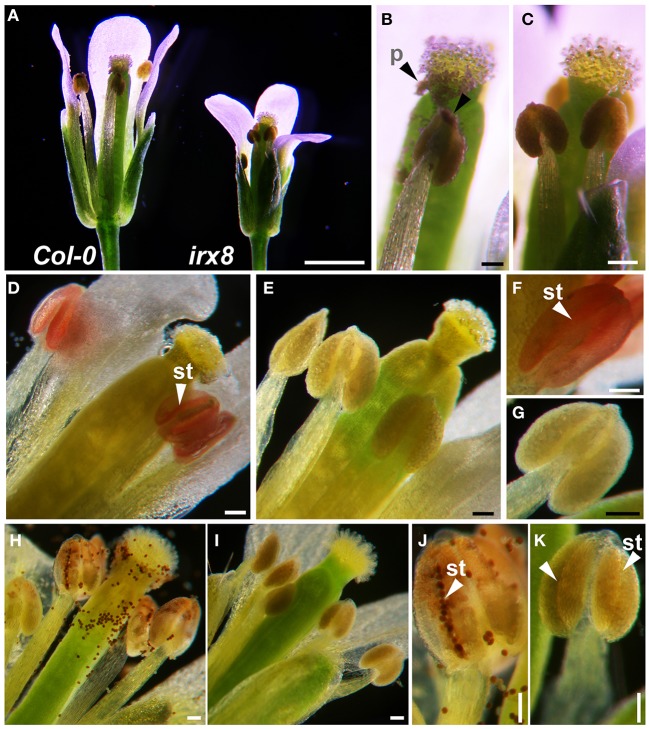
**Loss of lignin in anther endothecium cells results in failure of anther dehiscence in *irx8* mutants. (A)** Light microscope images of wild-type and *irx8* open flowers (stage 13, anthesis). **(B)** Close-up of *Col-0* pistil and anthers. **(C)** Close-up of *irx8-5* pistil and anthers. **(D–G)** Red phloroglucinol-HCl staining of flower anther indicates presence of lignin. Endothecium layer stains red in wild type **(D)**, the lack of staining in *irx8-5* **(E)**, close-up of a wild-type anther (stomium indicated by arrow, **F**), and close-up of an *irx8* anther showing no staining **(G)**. **(H–K)** Mäule staining of flowers of wild type: pollen and dehisced anther walls stained red **(H)**; lack of staining in *irx8-5* **(I)**; close-up of wild-type anther stomium (arrowhead), released pollen stained dark red **(J)**; close-up of *irx8-5* stomium (arrowhead), no lignin staining and no pollen released **(K)**. Bar in **A** = 1 mm; bar in **B–K** = 0.1 mm. p, pollen; st, stomium.

We investigated the cause of *irx8* sterility. First, we used a dissecting microscope to observe open flowers of *irx8* and wild type (WT) at stages 13 and 14 (anthesis and fertilization) (Bowman, 1994), respectively (Figure 1). An open flower of *irx8-5* contains reproductive organs of the correct shape but smaller in size than wild type (Figure 1A) and has shorter stamens (as previously described; Persson et al., 2007). At this stage, wild-type anthers have already dehisced and pollen grains are released onto the stigma and style (Figure 1B, arrowheads). The *irx8-5* mutant, however, has smooth intact anthers and shows no pollen release (Figure 1C). The same phenotype was also observed in the *irx8-2* mutant (Supplemental Figure S4).

**Figure 2:**
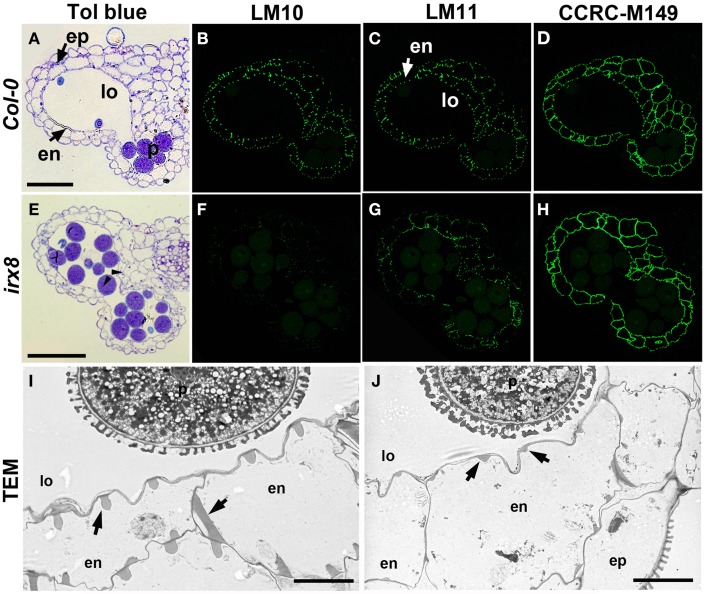
**Reduction in xylan deposition in anther endothecium cells in *irx8* mutants**. LR White-embedded WT **(A)** and *irx8-5* **(E)** anther transverse sections (250 nm-thick) stained with toluidine blue O (Tol blue), arrowheads point to septum breakage. WT **(B–D)** and *irx8* **(F–H)** anthers immunolabeled with xylan-reactive antibodies LM10 **(B,F)**, LM11 **(C,G)**, and CCRC-M149 **(D,H)**. Bar = 50 μm for **(A–H)**. TEM of WT **(I)** and *irx8-5* **(J)** anther. Arrows point to the secondary wall thickenings in the endothecium cell layer, which is reduced in *irx8-5*. ep, epidermis; en, endothecium; p, pollen; lo, locule. Bar = 5 μm for **(I)** and **(J)**.

**Figure 3:**
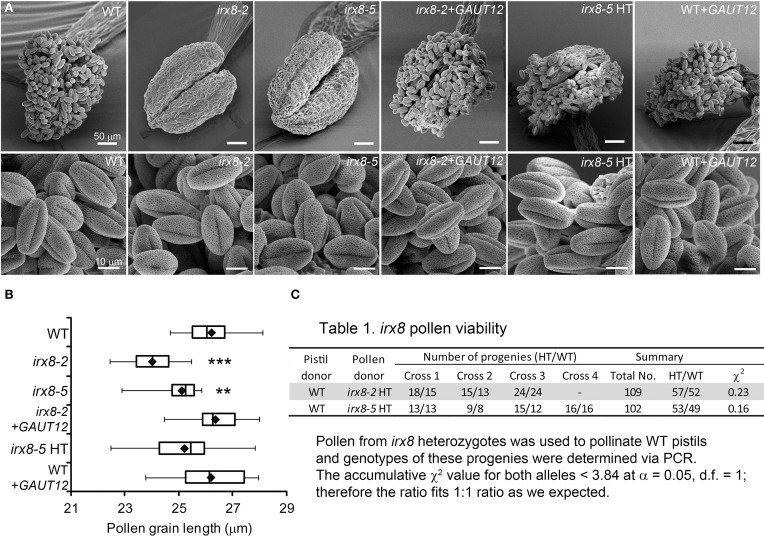
**SEM of anthers and pollen grains (PG) from *irx8* and *GAUT12*-transgenic plants. (A)** SEM images of anthers and PG from open flowers of WT, *irx8-2*, *irx8-5*, *irx8-2*+*GAUT12*, *irx8-5* heterozygote (HT), and WT+*GAUT12*. Bar = 50 μm in upper panel and = 10 μm in lower panel. **(B)** Box plot diagram of PG length measured under SEM of WT (*n* = 10), *irx8-2* (*n* = 19), *irx8-5* (*n* = 21), *irx8-2*+*GAUT12* (*n* = 20), *irx8-5* HT (*n* = 26), and WT+*GAUT12* (*n* = 25). *n*, number of PG measured. The One-Way ANOVA was significant and a Bonferroni corrected *post-hoc t*-test indicates that PG length is significantly smaller in *irx8-2* and *irx8-5* and is complemented by *GAUT12*. The average pollen size of irx8 HT and WT+*GAUT12* were similar to the size of WT pollen. *P*_t-test_ < 0.01 (^**^), and < 0.001 (^***^). **(C)** The *irx8* pollen viability test. Pollen from heterozygous *irx8* plants was used to pollinate WT pistils and the progeny were genotyped by PCR. The accumulative χ^2^ value based on the total number of progeny was calculated for both alleles. Both were smaller than 3.84, which is the critical value for χ^2^ distribution at α = 0.05 and d.f. = 1. The results showed that the HT vs. WT progeny fit the 1:1 ratio according to the Mendelian law of segregation.

Secondary wall thickening in endothecium cells of pollen sacs provides the mechanical force for anther dehiscence. After anther dehydration and pollen swelling, the stomium breaks open to release pollen at anthesis, followed by anther filament extension to achieve fertilization (Wilson et al., 2011). Phloroglucinol-HCl staining (Wiesner test), which colorimetrically identifies coniferaldehyde end-groups in G lignin, and Mäule reagent, which reacts with syringylpropane moieties of S lignin to produce a rose red color (Lewis and Yamamoto, 1990) were used to compare lignification in the wild type and mutant. The lignin of the secondary wall thickening along the stomium furrow in wild-type mature anther stained red using phloroglucinol-HCl (Figures 1D,F, arrowheads). In contrast, the stomium furrow was not stained at all in *irx8* anthers at the same stage (Figures 1E,G), suggesting a reduction of G lignin in the *irx8* endothecium cell layer. In addition, released pollen from the wild type stained brownish-red with Mäule reagent at the stomium opening (Figure 1H). The wild-type pollen sacs were also partially stained since the stain was able to access the endothecium layer through the open stomium (Figure 1J). However, at the same developmental stage (anthesis), such staining was absent in *irx8* anthers (Figures 1I,K). Despite no pollen release in *irx8* flowers, the anthers appeared to be turgid (Figure 1C; Supplemental Figure S4), suggesting that the pollen inside was swollen. The enzymatic lysis of the *irx8* stomium and septum breakage also occurred in the anther, as seen in transverse sections (Figure 2E, black arrowheads). The indehiscent anther phenotype was confirmed in both the *irx8-2* and *irx8-5* mutants using SEM. This phenotype was complemented by the *GAUT12-EGFP* construct (Figure 3A; Supplemental Figure S4).

The secondary wall thickening of the endothecium cells (Figure 2A) contains not only lignin, but also xylan and cellulose (Wilson et al., 2011). Since *irx8* has a known xylan defect, we fixed, embedded, and sectioned open flowers of *irx8* and analyzed anther cell walls in the sectioned tissues using plant cell wall glycan-directed monoclonal antibodies against xylan and other major polysaccharides (Pattathil et al., 2010). In wild-type endothecium cell walls, LM10 (Figure 2B) which binds low-substituted xylan and LM11 (Figure 2C) which binds both low- and high-substituted xylan (McCartney et al., 2005) showed almost identical punctate labeling patterns, demonstrating a common location of the two xylan epitopes recognized by these two antibodies. Both epitopes are reduced in *irx8* (Figures 2F,G), with LM10 labeling almost completely absent in *irx8* (Figure 2F), indicating a pronounced reduction in low-substituted xylan recognized by LM10. Transmission electron microscopy (TEM) showed that the secondary wall thickening in wild-type endothecium cells, in the shape of teeth- and ribbon-like structures (Figure 2I, arrows), is significantly reduced in *irx8* endothecium cells (Figure 2J, arrows). Xylan present in the *irx8* endothecium was detected using CCRC-M149 (Figure 2H), CCRC-M137, CCRC-M138, and CCRC-M160 antibodies (Supplemental Figures S5I,M,N), suggesting that *GAUT12* affects some xylan synthesis in endothecium cells and that some xylan epitopes remain unchanged in *irx8* anthers compared to wild type (Figure 2D, Supplemental Figures S5B,F,G). No difference was seen in *irx8* endothecium walls using anti-pectin antibodies JIM5, JIM7, and CCRC-M38 (Supplemental Figures S5U–W) that recognize HG epitopes with various degrees of methylesterification, and little change was observed in this tissue using antibodies CCRC-M14, JIM13, and CCRC-M1, which recognize RG-I backbone, arabinogalactan protein (AGP), and fucosylated xyloglucan epitopes, respectively (Supplemental Figures S5X–Z).

TEM revealed smaller pollen size in *irx8-5* (Figure 2J), which was confirmed by SEM (Figure 3A). While the wild-type pollen grains are uniform in size and shape, both mutant alleles of *irx8* have smaller pollen grains when manually released from pollen sacs (Figure 3B). Occasionally we observed defective *irx8* pollen grains under both SEM and TEM. However, since pollen development is easily affected by growth conditions, and pollen formation involves both sporophytic and gametophytic factors (Ariizumi and Toriyama, 2011), we wanted to test directly whether *GAUT12* affects pollen fertility. We found that manually released *irx8* pollen was able to pollinate both wild-type and heterozygous *irx8* pistils, and wild-type pollen was able to pollinate the *irx8* pistil, both producing viable seeds. Interestingly, however, manual fertilization of the homozygous *irx8* pistil with its own pollen was not successful. We observed that the *irx8* inflorescence often dried soon after the stem stopped elongating, which may be due to the poor water conduction in this mutant. This may explain why manual fertilization of the *irx8* pistil was unsuccessful.

We used quantitative PCR to determine if *GAUT12* is expressed in pollen. Small amounts of *GAUT12* transcript was detected in hydrated pollen grains and pollen tubes (Supplemental Figure S6). However, no expression of *CESA4* or *IRX9* transcript, genes known to encode secondary wall cellulose and xylan biosynthetic proteins, respectively (Taylor et al., 2003; Peña et al., 2007), was detected in either tissue. In contrast, relatively high levels of expression were observed for *GAUT1* and *CESA1* (Supplemental Figure S6B), genes known to be involved in primary cell wall pectin and cellulose biosynthesis, respectively (Arioli et al., 1998; Sterling et al., 2006; Atmodjo et al., 2011).

To further analyze whether *GAUT12* affects pollen tube formation and viability, we manually fertilized wild-type pistils with pollen from the *irx8* heterozygote, a pollen population consisting of both *irx8* and wild-type pollen produced in equal amounts. The number of heterozygote vs. WT progeny from these crosses were 57:52 and 53:49 for *irx8-2* and *irx8-5* heterozygotes, respectively, fitting the 1:1 ratio expected for Mendelian segregation according to a χ^2^ test (Figure 3C). This result demonstrated that *irx8* pollen has normal fertility. From these results we conclude that the sterility of *irx8* mutants is due to indehiscent anthers and to the resulting inability of the mutant to release pollen for fertilization.

### The *irx8* mutant has low lignin content in basal stems and reduced expression of major lignin biosynthetic genes

**Figure 4:**
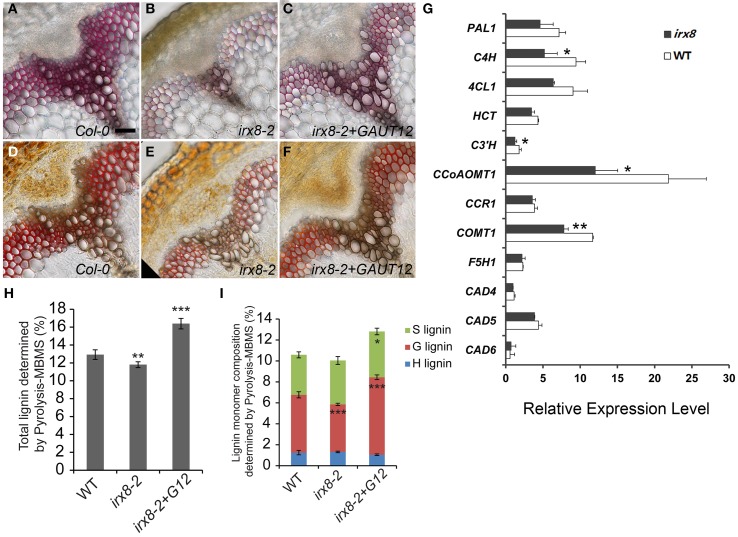
**Measurement of lignin and lignin biosynthetic gene expression in the *irx8* mutant**. Phloroglucinol-HCl staining of free-hand basal stem cross-sections of WT **(A)**, *irx8-2* **(B)**, and *irx8-2*+*GAUT12* **(C)**. Mäule staining of free-hand basal stem cross-sections of WT **(D)**, *irx8-2* **(E)**, and *irx8-2*+*GAUT12* **(F)**. Bar = 50 μm for **A–F**. **(G)** Expression analyses in Arabidopsis WT and *irx8* lower stems of lignin biosynthetic genes using Real-Time PCR. Genes labeled with asterisks have significantly lower expression in *irx8-5* compared to WT (^*^p < 0.05; ^**^p < 0.01). The relative expression level of each gene was normalized using *Actin2* as the reference gene and the expression of *C3′H* in wild-type basal stem was set to 1. Values are mean ± standard deviation (*n* = 3). Lower-stem refers to the lower half of the inflorescence. PAL, phenylalanine ammonia lyase; C4H, trans-cinnamate 4-hydroxylase; 4CL, 4-coumarate: CoA ligase; HCT, hydroxycinnamoyl-CoA: shikimate/quinate hydroxycinnamoyltransferase; C3′H, 4-coumaroyl shikimate 3′-hydroxylase; CCoAOMT1, caffeoyl-CoA 3-*O*-methyltransferase; CCR, cinnamoyl-CoA reductase; F5H, ferulate 5-hydroxylase; COMT, caffeic acid/5-hydroxyferulic acid *O*-methyltransferase; CAD, cinnamyl alcohol dehydrogenase; PER/LAC, peroxidases/laccases. **(H)** Total lignin (%) determined by pyrolysis molecular beam mass spectrometry (pyMBMS) of stem alcohol insoluble residues. **(I)** Lignin monomer composition determined by pyMBMS. Lignin content percentage (%) was corrected by equivalence to Klason lignin. The *irx8-2* and *irx8-2+GAUT12* values were compared to WT as determined by ANOVA and *post-hoc t*-tests with Bonferroni correction (^*^p < 0.05; ^**^p < 0.01; ^***^p < 0.001; *n* = 6) for **(H,I)**.

Reduction of lignin in the *irx8* endothecium cell layer led us to examine the lignin content in *irx8* stems by histochemical staining. We found a reduction in total lignin compared to wild type (Figure 4), an observation recently confirmed by Petersen et al. (2012). Wild-type xylem vessels and fibers had thick secondary walls with evenly distributed lignin (Figures 4A,D). In contrast, interfascicular fiber cells of *irx8* mutants stained very weakly with phloroglucinol-HCl (Figure 4B), indicating a loss of G lignin. Staining with Mäule reagent revealed a brown staining in *irx8* xylem cells and reduced red staining in the interfascicular fiber cells (Figure 4E), indicative of a possible but lesser reduction in S lignin deposition. The lignin content was recovered in the *GAUT12-*complemented *irx8 (irx8+GAUT12)* plants, as shown by staining using both stains (Figures 4C,F).

To determine whether the reduced lignin content was due to reduced biosynthesis of lignin monomers, we quantified the steady state levels of transcripts for 10 enzymes of the lignin biosynthetic pathway (Raes et al., 2003) in the basal half of *irx8* stems (Figure 4G). Significantly reduced expression was observed for several key enzymes including trans-cinnamate 4-hydroxylase (C4H), 4-coumaroyl shikimate 3′-hydroxylase (C3′H), caffeoyl CoA *O*-methyltransferase (CCoAOMT1), and caffeic acid/5-hydroxyferulic acid *O*-methyltransferase (COMT1). We conclude that the generation of lignin precursors is likely down-regulated in the Arabidopsis *irx8* mutant.

To further evaluate lignin structure and lignin composition in *irx8* stems, AIR was analyzed using pyrolysis molecular beam mass spectrometry (pyMBMS) (Sykes et al., 2008). There was a moderate but significant reduction (9%) in total lignin which was complemented by the *GAUT12-EGFP* transgene (Figure 4H). The reduction of total lignin in *irx8* stems was largely due to a lower amount (18% reduction) of guaiacyl (G) subunits, whereas there was a relatively normal amount of *p*-hydroxyphenyl (H) and syringyl (S) subunits in *irx8* (Figure 4I). The reduction in G lignin resulted in an increased S/G ratio (*irx8-2*: 0.9 ± 0.09; WT: 0.7 ± 0.06; *P*_t-test_ = 0.0006). The *irx8+GAUT12* plants had an S/G ratio of 0.6 ± 0.04 (*P*_t-test_ = 0.011), slightly lower than that of the wild type.

**Figure 5:**
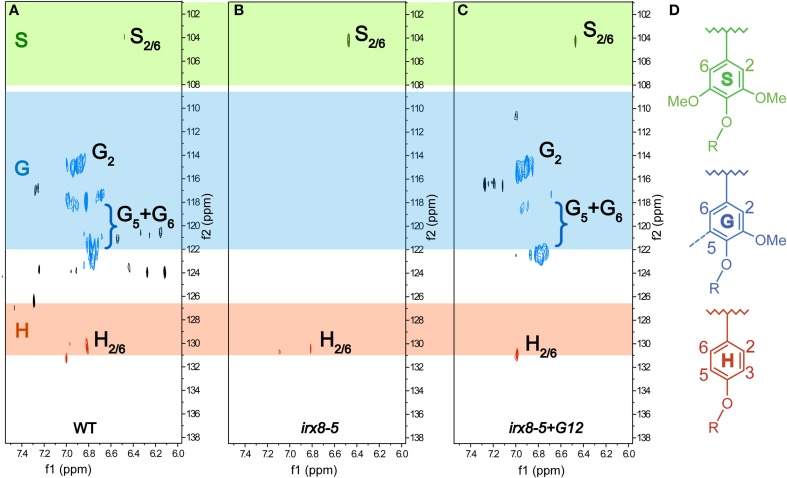
**Aromatic regions of 2D ^13^C-^1^H Heteronuclear Single-Quantum Correlation (HSQC) NMR spectra**. Chlorite extracts prepared from WT **(A)**, *irx8-5* **(B)**, and *irx8-5*+*GAUT12* **(C)** stem alcohol insoluble residues (AIR) are shown. The ^13^C/^1^H signals for G lignin were absent in the HSQC spectrum of *irx8-5* **(B)** compared to wild type **(A)**, while the G lignin signals were recovered in the spectrum of *irx8-5+GAUT12* **(C)**. **(D)** Lignin monomer structures.

We further characterized the lignification in *irx8* and wild-type stems by sequentially extracting stem AIR with 50 mM ammonium oxalate, 50 mM sodium carbonate, 1 M and 4 M KOH, acidified sodium chlorite, and post-chlorite 4 M KOH. We first used 2D ^13^C-^1^H HSQC NMR spectroscopy to analyze the cell wall fraction extracted with acidified sodium chlorite, which de-lignifies the biomass. The aromatic region in the HSQC spectrum of the *irx8* chlorite extract revealed a dramatic loss of G lignin C/H-2, 5, 6 signals (Figure 5B) compared to that of the wild-type chlorite extract (Figure 5A). This result is consistent with the reduction in G lignin identified by phloroglucinol-HCl staining (Figure 4B) and pyMBMS (Figure 4I). Consistent with the complementation result (Supplemental Figures S1, S2), G lignin cross peaks were present in the HSQC spectrum in the chlorite extract of *irx8+GAUT12* complemented plants (Figure 5C). Only trace amounts of H and S lignin signals were identified in the chlorite extract across all three samples, indicating that either these monomers were lost during sample preparation or located in other wall extracts.

**Figure 6:**
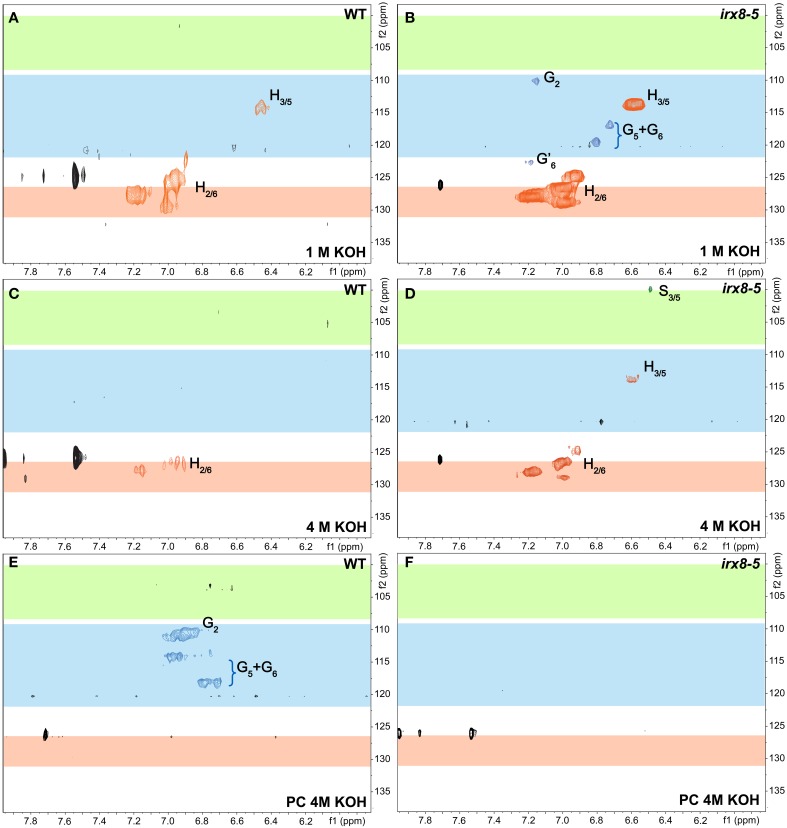
**Aromatic regions of 2D ^13^C-^1^H Heteronuclear Single-Quantum Correlation (HSQC) NMR spectra of 1 M KOH, 4 M KOH, and post-chlorite-4 M KOH extracts of WT and *irx8-5* mutant walls**. WT **(A)** and *irx8-5* **(B)** 1 M KOH extracts. WT **(C)** and *irx8-5* **(D)** 4 M KOH extracts. WT **(E)** and *irx8-5* **(F)** post-chlorite 4 M KOH extracts. The signals of H, S, and G lignin monomers are as labeled.

We thus used 2D ^13^C-^1^H HSQC NMR spectroscopy to examine all other wall extracts from both wild-type and *irx8* stems, including ammonium oxalate-, sodium carbonate-, 1 M KOH-, 4 M KOH-, post-chlorite 4 M KOH (PC4MKOH) fractions, and residual pellets. Surprisingly, we found that both the 1 M and 4 M KOH extracts of wild type and *irx8* contained most of the H lignin, with the major H lignin signals located in the 1 M KOH extract (Figures 6A–D). The G lignin in the *irx8* mutant, although significantly reduced in amount, was found almost exclusively in the 1 M KOH extract (Figure 6B), while the G lignin in the wild type was located in both the chlorite- (Figure 5A) and PC4MKOH extracts (Figure 6E). These results reveal that the G lignin in the *irx8* mutant is more easily extracted than in the wild type. Only trace amounts of S lignin were found across all five wall extracts, indicating that S lignin was either composed of small molecules or degraded to small molecules during sequential extractions, and thus lost during sample dialysis. The aromatic regions of wild-type and *irx8* pectin-enriched fractions, i.e., ammonium oxalate- and sodium carbonate-extracts, showed comparable amounts of H lignin. The cellulose-enriched pellets did not show recognizable signals for major lignin structures (Supplemental Figure S7). Compared to wild type, the lignin aliphatic (side-chain) regions in the HSQC NMR spectra of the *irx8* chlorite extract suggested a reduction in the signals for β-*O*-4, β-5, and β −β linked lignin (Supplemental Figure S8B), which were calculated by density functional theory to be the major and thermodynamically favorable linkages within lignin polymers (Sangha et al., 2012), and hence the most stable during acidic chlorite extractions.

### Glycome profiling indicates altered extractability of xylan, pectin, and AG epitopes in walls of the *irx8* mutant

**Figure 7:**
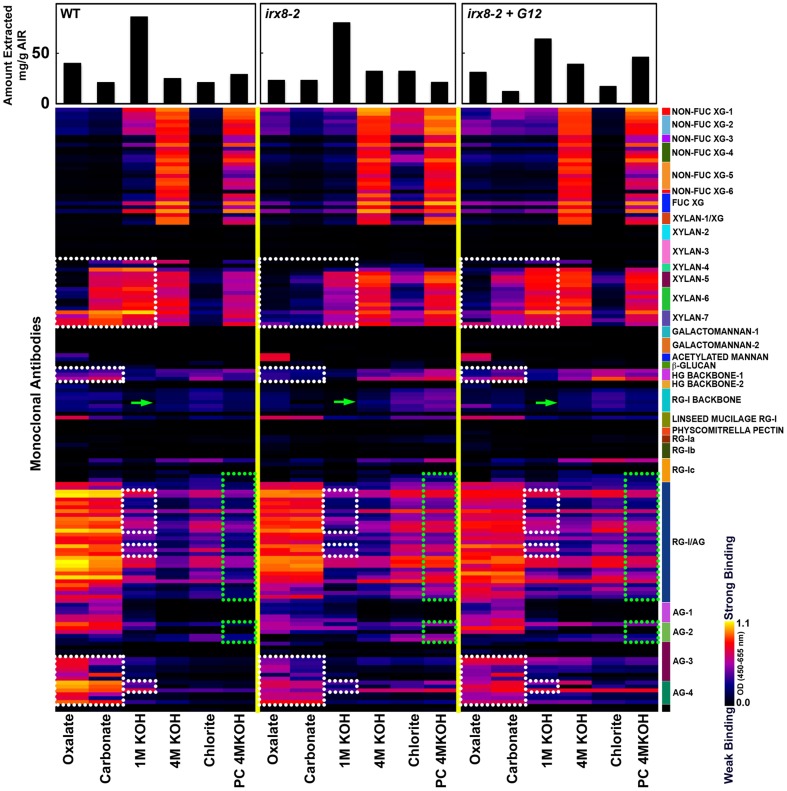
**Glycome profiling of cell walls prepared from 7/8-week-old stem tissues of *irx8-2*, *irx8-2+GAUT12*, and wild-type (WT) plants**. Sequential cell wall extracts were made from respective cell wall preparations using ammonium oxalate (oxalate), sodium carbonate, potassium hydroxide (1 M and 4 M KOH), acidified chlorite and potassium hydroxide post chlorite (PC4MKOH). The resulting extracts were screened by ELISA using a suite of 155 monoclonal antibodies (mAbs) directed against diverse epitopes present on major plant cell wall glycans (See Supplemental Table S2). The ELISA binding responses are represented as heatmaps with white-red-black scale indicating the signal strength of ELISA (white, red, and dark-blue colors depict strong, medium, and no binding, respectively). The mAbs are grouped based on their specificity for cell wall glycans as shown in the panel at right hand side of the figure. The actual amount of material extracted with each extraction reagent is depicted in bar graphs at the top of the heatmaps. Glycan epitopes with reduced signals in *irx8* are outlined with white dotted blocks, while those with increased signals in *irx8* are outlined with green dotted blocks. Green arrows point to CCRC-M14 labeling.

To examine cell wall glycan epitope changes associated with the loss of *GAUT12* function and to correlate *irx8* wall polysaccharide epitope changes with the lignin signal alterations observed by NMR, we performed glycome profiling analyses on wall extracts from wild type, *irx8*, and *GAUT12-*complemented *irx8* (*irx8*+*GAUT12*) stems. The analyses correlated ELISA signals of different monoclonal antibody (mAb) groups with carbohydrates released in each fraction. Cell walls (i.e., AIR) prepared from *irx8* showed major differences in their glycome profiles when compared with WT walls. These differences are highlighted by dotted blocks in Figure 7.

A pronounced difference was noted in the extractability of xylan epitopes in *irx8*, compared to the wild type. Ammonium oxalate-, sodium carbonate-, and 1 M KOH-extracts prepared from *irx8* walls contained significantly less xylan epitopes recognized by the xylan 4 through 7 groups of xylan-directed mAbs. Since less or similar amounts of carbohydrate mass was isolated in these extracts from *irx8* walls compared to wild-type walls, there was significantly less xylan in the oxalate-, carbonate-, and 1 M KOH extracts in *irx8* (Figure 7). The results indicate a loss of easily extractable xylan that is potentially associated with pectin in *irx8*, since these wall extracts contain large amounts of pectin. Both the 4 M KOH- and PC4MKOH extracts of *irx8*, however, displayed a marginal increase in binding of xylan mAbs compared to the corresponding WT extracts. The result that more xylan in the *irx8* mutant was extracted under harsher conditions (i.e., with 4 M KOH and post-chlorite 4 M KOH) correlates with the previous finding that the 4 M KOH fractions from *irx8* stems contain some xylan of higher molecular weight than the WT counterparts (Brown et al., 2007; Peña et al., 2007).

Other differences in the glycome profile of the *irx8* mutant include a reduced presence of HG backbone epitopes (those recognized by HG Backbone-I group of mAbs, Figure 7) and of arabinogalactan (AG) epitopes in the oxalate and carbonate extracts (those recognized by AG-3 and AG-4 groups of mAbs, Figure 7), as well as a reduction in pectic arabinogalactan epitopes (recognized by RG-I/AG antibodies) and AG (those recognized by the AG-4 group of mAbs) in the 1 M KOH extract (Figure 7). These results indicate a concomitant loss of pectin, AG, and xylan in these wall extracts. The complemented line *irx8+GAUT12* shows a partial reversion of the glycome profile pattern to that of the WT, including an enhanced extractability of xylan, HG, and AG epitopes in the oxalate and carbonate extracts. The chlorite extracts of *irx8* showed significantly enhanced levels of hemicellulose epitopes including xylan and xyloglucan (Non-Fuc XG-1 to XG-6, Fuc-XG, and xylan-1/XG) compared to those of wild type and *irx8+GAUT12*. There was also a marginal increase in binding of antibodies against pectic arabinogalactan (RG-I/AG and AG2) in the *irx8* chlorite extract. This wall extract contained more mass in the *irx8* extract compared to the wild type, suggesting an increased weight ratio of carbohydrates to lignin in this extract (possibly caused by the reduction of lignin content described above; Figure 5B). An increase in pectic arabinogalactan (RG-I/AG and AG2) epitope content was also observed in the *irx8* PC4MKOH extract, which contained less mass compared to the wild type and *irx8+GAUT12*. These results indicate a shift in the extractability of RG-I/AG epitopes in the *irx8* mutant that may compensate for the slight reduction of these epitopes in the oxalate- and carbonate fractions. Together, these results show that *irx8* has significant changes in the extractability of glycan epitopes, particularly of xylan, pectin, and some AG.

### The *irx8* mutant exhibits increased RG-I labeling and altered xylan localization patterns in fiber cell walls

**Figure 8:**
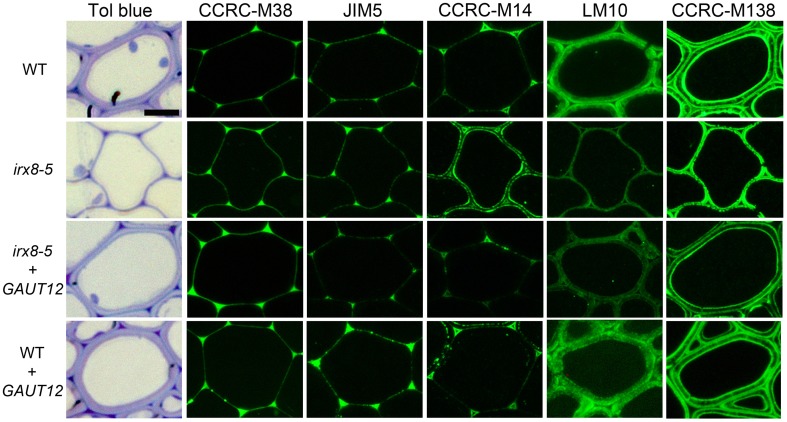
**Immunolabeling of interfascicular fiber cells from basal stems of 6-week-old wild-type (WT), *irx8-5*, *irx8-5+GAUT12*, and WT+*GAUT12* plants with anti-pectin and anti-xylan antibodies**. CCRC-M38 recognizes un-esterified HG, JIM5 recognizes low-esterified HG, CCRC-M14 recognizes RG-I backbone, LM10 (Xylan-6) binds to low-substituted xylan, and CCRC-M138 (Xylan-6) binds to unsubstituted xylopentaose (Pattathil et al., 2010). Bar = 10 μm.

In addition to the significant reduction in xylan in *irx8* fiber cell walls based on reduced immunolabeling by anti-xylan antibodies LM10 and LM11 (Figure 8; Supplemental Figures S3F, S8), there was also an unexpected increase in immunolabeling by the antibody CCRC-M14, which recognizes an RG-I backbone epitope (Figure 8). CCRC-M14 binds to an empty triangle-shaped region in tricellular junctions of WT fiber cells (Figure 8). However, in 6–7-week-old *irx8* basal stems, this antibody also labels the inner layer of fiber cell walls in a dotted and lamellate pattern, suggesting possible increases in the amount or accessibility of RG-I-associated cell wall epitopes in *irx8* fiber cells. Consistent with this result, a slight increase in CCRC-M14 labeling was also observed in the glycome profiles in the 4 M KOH-, chlorite-, and PC4MKOH extracts of *irx8* compared to the WT and *irx8+GAUT12* counterparts (Figure 7, green arrows). It is worth mentioning that we have also observed the loss of the CCRC-M14 labeling pattern in fiber cells in 8-week-old (or older) WT and *irx8* basal stem sections, indicating that this phenotype may relate to a specific developmental stage. The *GAUT12-EGFP* construct complemented the CCRC-M14 phenotype in *irx8* fiber cells (Figure 8). Over-expression of *GAUT12* in WT (WT*+GAUT12*) plants, on the other hand, led to an occasional accumulation of CCRC-M14 reactive material in areas of the wall outside the triangular cell corner regions observed in the WT (Figure 8). There was, however, no obvious growth phenotype or altered sugar composition in WT vs. *GAUT12* over-expression lines (Supplemental Figures S1G, S3D,H, S4, S10).

We applied a selection of six xylan-directed antibodies to basal stem sections of wild type, *irx8*, and *GAUT12*-transgenic lines (Supplemental Figure S9). Compared to WT fiber cells, there is a reduction in the labeling intensity in *irx8* fibers with LM10, CCRC-M137, and CCRC-M160. LM10 binds to low-substituted xylan (McCartney et al., 2005), while both CCRC-M138 and CCRC-M160 bind to unsubstituted xylopentaose in ELISAs. The fiber walls of the *irx8+GAUT12* plant are much thicker than those of *irx8*, but are still slightly thinner than WT fiber walls and show lower labeling intensities with LM10, LM11, and CCRC-M137, a pattern resembling those of the *irx8* mutant labeled using these three antibodies (Supplemental Figure S9). Interestingly, we noticed a double-ring labeling pattern in WT fiber cell walls labeled with CCRC-M138, a monoclonal antibody that recognizes unsubstituted xylopentaose (Figure 8). In WT fiber cells, both an inner, plasma membrane-proximal wall domain and an outer, middle lamella-proximal wall domain are solidly and continuously labeled with CCRC-M138, whereas xylan between these two rings, the middle layer, is mostly not labeled with this antibody. In *irx8* fibers the CCRC-M138-labeled double-ring, while still present as can be observed readily in cell corners, has collapsed and displays a discontinuous (dotted) pattern, which is clearly different from that of WT fiber walls. The reduced thickness or loss of the middle layer in the *irx8* mutant results in much thinner fiber cell walls. Overall, the *GAUT12* construct complemented the CCRC-M138 phenotype in *irx8* fiber cells (Figure 8), although the labeling intensity in the labeled wall domains appears slightly uneven (Supplemental Figure S9). CCRC-M160 shows a very similar labeling pattern to CCRC-M138 in the *GAUT12*-complemented (*irx8+GAUT12*) line, albeit its double-ring pattern is less manifest in WT and WT+*GAUT12* fibers (Supplemental Figure S9). The xylan deposition changes were also reflected in the altered xylan extraction patterns identified in *irx8* by glycome profiling (Figure 7).

### Immunoabsorbed GAUT12 is not an HG:GALAT with characteristics comparable to GAUT1

**Figure 9:**
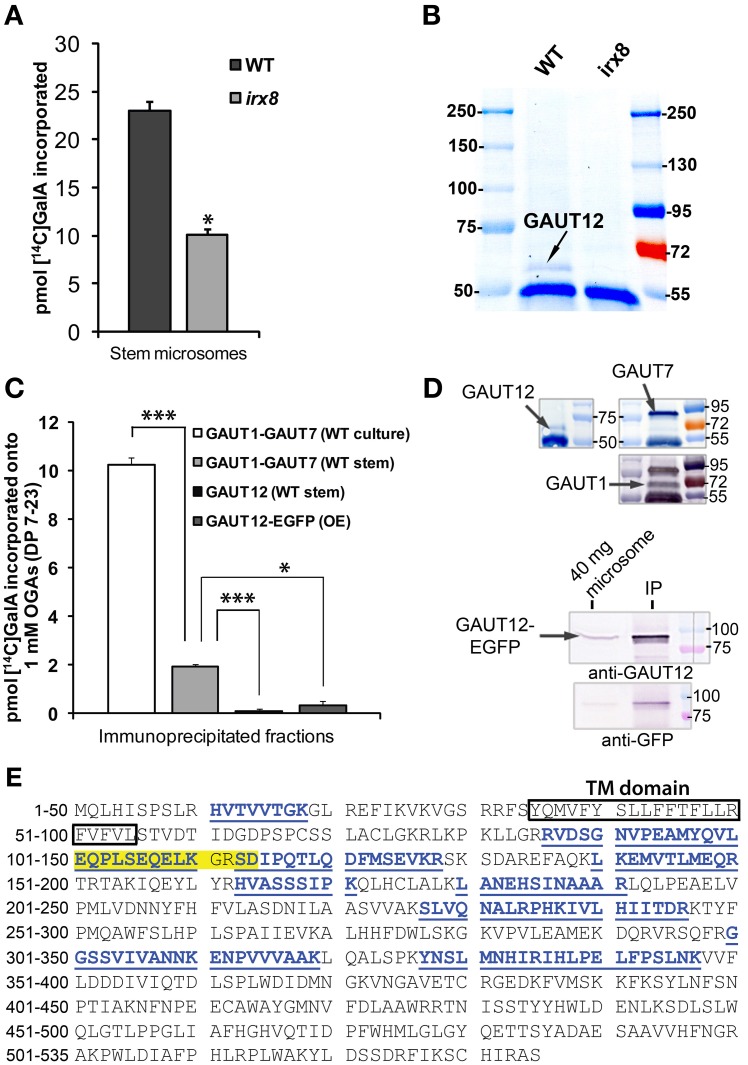
**Enzymatic activity assays for GAUT12 and the anti-GAUT12 antibody. (A)** HG:GalAT activity of total microsomes (50 μg) from WT and *irx8-5* stems. Value = mean ± standard deviation (*n* = 3), ^*^indicates significant reduction of HG:GalAT activity in *irx8* stem microsomes at *P*_(t-test)_ < 0.05. **(B)** Western blot analysis of immunoprecipitated (IP) GAUT12 fractions from wild-type (WT) and *irx8-5* 7-week-old stem microsomes (500 μg total protein) using 30 μl anti-GAUT12 antibody-conjugated magnetic beads. GAUT12 protein band as indicated (~58–60 kDa) is present in WT but absent in *irx8-5* microsomes. **(C)** HG:GalAT activity of immunoabsorbed-GAUT12 from WT stem microsomes and from GAUT12-EGFP fusion protein expressed in Arabidopsis suspension culture cells (OE). Immunoabsorbed-GAUT1:GAUT7 complex from WT suspension cell cultures and from WT stem microsomes were used as positive controls. Equal amounts of the corresponding antibody-conjugated beads (30 μl) were incubated with equal amounts of TX-100 permeabilized microsomes from different tissues (500 μg total protein). Value = mean ± standard deviation (*n* = 3), ^*^, ^***^ indicates significant reduction of HG:GalAT activity determined by One-Way ANOVA and *post-hoc* Bonferroni corrected *t*-test at *p* < 0.05 and *p* < 0.001, respectively. Immunoabsorbed-GAUT12 and GAUT12-EGFP activity are similar to background with *P*_(t-test)_ values = 0.44 and 0.22, respectively. All enzyme activity assays were repeated at least twice. One set of representative results is shown. **(D)** Western blots showing the presence of GAUT12, GAUT1, and GAUT7 in the corresponding immunoabsorbed fractions from WT stem tissues. GAUT12 was immunoabsorbed by the anti-GAUT12 antibody, and the GAUT1:GAUT7 complex was immunoabsorbed by the anti-GAUT7 antibody. Arabidopsis culture expressed-GAUT12-EGFP (~86 KDa) fusion protein in the anti-GAUT12 immunoabsorbed fractions used in reactions in **(C)** shown in the lower panel. **(E)** GAUT12 protein sequence (535-a.a.). The antigenic-peptide used to generate the anti-GAUT12 antibody is highlighted in yellow. Peptides identified in GAUT12-IP fractions by LC-MS/MS are labeled in blue with underlines. Boxed sequence indicates the GAUT12 transmembrane (TM) domain predicted by TmHMM_v2.

The increased CCRC-M14 labeling in *irx8* stems which suggested a change in RG-I, along with the previously reported reduction in a subfraction of HG (Persson et al., 2007), is consistent with the hypothesis that GAUT12 functions as an HG:GalAT required for secondary wall and xylan formation. To test this possibility, we measured HG:GalAT activity in microsomes from *irx8* stems which were shown to contain no GAUT12 protein (Figure 9B). The *irx8* microsomes contained 55% of the GAUT1-like HG:GalAT activity present in wild-type microsomes (Figure 9A). However, it remained unclear whether this reduction was directly due to the loss of GAUT12 activity or due to indirect effects on other enzymes, such as the HG:GalAT GAUT1, which is known to be expressed in Arabidopsis stems (Atmodjo et al., 2011). To directly test if GAUT12 has HG:GalAT activity, we generated a polyclonal anti-GAUT12 antibody that specifically recognizes GAUT12 and does not cross-react with GAUT1 or GAUT7 (Figures 9D,E; Supplemental Figure S11). LC-MS/MS was used to verify the specificity of the anti-GAUT12 antibody because the GAUT protein family contains 15 members with high sequence identity and similarity (Sterling et al., 2006). LC-MS/MS showed that peptides recovered from the protein immunoprecipitated by anti-GAUT12 are GAUT12-specific sequences and not those belonging to other GAUT proteins (Figure 9E). The antigen-purified anti-GAUT12 antibody was used to immunoabsorb-GAUT12 from detergent-permeabilized microsomes from wild-type stems (Figure 9D), and the immunoabsorbed GAUT12 was assayed for HG:GalAT activity. Neither the immunoabsorbed-GAUT12 from wild-type (WT) stem microsomes nor the immunoabsorbed-GAUT12-EGFP from Arabidopsis suspension culture cells over-expressing the GAUT12-EGFP fusion protein showed significant HG:GalAT activity (Figure 9C), although both proteins were confirmed to be present in these fractions by western blotting (Figure 9D). Thus, GAUT12 either does not have HG:GalAT activity, or its HG:GalAT activity is biochemically distinct from that of GAUT1 and cannot be assayed under the same reaction conditions.

## Discussion

### GAUT12 is required for anther dehiscence

Persson et al. (2007) have described the *irx8* mutants as semi-sterile (*irx8-1* and *irx8-2*) having shorter anther filaments, less pollen than the wild type, and no seeds (Persson et al., 2007). Indeed, in our hands we were not able to recover any seeds from either *irx8-2* or *irx8-5* plants. Other dwarf mutants with collapsed xylem phenotypes, particularly *irx9* and *parvus-3*, have dehiscent anthers that release pollen (Supplemental Figure S4) and produce seeds under the same growth conditions. It is believed that *IRX9* is involved in xylan backbone elongation and *GAUT12* together with *PARVUS/GATL1* are involved in XRES biosynthesis (Brown et al., 2007; Lee et al., 2007a,b; Peña et al., 2007). Our results suggest that the function of *GAUT12* may, at least in the endothecium cell layer that is critical for anther dehiscence, be distinct from that of *IRX9* and *PARVUS/GATL1* in regards to xylan and lignin synthesis and deposition. Alternatively, anther dehiscence in both the *irx9* and *parvus-3* mutants may be due to expression of functionally redundant genes in the endothecium cell layer.

The reduction of lignin in *irx8* anther cell walls (Figures 1E,I), together with the reduction in xylan as recognized by LM10 and LM11 in endothecium cell walls (Figures 2F,G), contributes to a lack of secondary wall thickening in the *irx8* endothecium layer (Figure 2J). Consequently, relatively low tension during anther wall dehydration leads to indehiscent anthers in *irx8* mutants. The pollen grains produced by *irx8*, albeit smaller in size (Figure 3B), are viable upon manual release and able to fertilize both wild-type and *irx8* heterozygote pistils. Furthermore, manual fertilization of wild-type pistils with *irx8* heterozygote pollen demonstrated that *irx8* pollen has similar viability to wild-type pollen (Figure 3C). Thus, the function of GAUT12 is not essential for pollen viability and fertilization. Prior published transcriptomic and proteomic analyses of pollen and pollen tubes have not detected *GAUT12* transcript or protein in these tissues (Wang et al., 2008; Zou et al., 2009). However, using quantitative RT-PCR we detected low *GAUT12* expression in hydrated pollen grains and elongating pollen tubes (Supplemental Figure S6), suggesting a potential role of GAUT12 in pollen, perhaps associated with pollen size (Figure 3B).

### Lack of GAUT12 function results in reduced amounts and altered extractability of G lignin in arabidopsis stem

We identified a reduction of lignin in the *irx8* mutant and used immunohistochemical staining (Figures 4B,E), pyMBMS (Figures 4H,I), and 2D ^13^C-^1^H HSQC NMR spectroscopy (Figure 5) to characterize this mutant phenotype in detail as well as study the possible connections between lignin and xylan deposition. In the semi-quantitative 2D ^13^C-^1^H HSQC NMR analyses, only trace amounts of H and S lignin were found in chlorite extracts from the WT, *irx8-5*, and *irx8-5+GAUT12* (Figure 5). H lignin signals, however, were identified in the oxalate-, carbonate-, 1 M and 4 M KOH extracts of both WT and *irx8*, with the major signals located in the 1 M KOH extracts (Figure 6; Supplemental Figure S7). These results suggest that H lignin is present in the pectin and hemicellulose-enriched wall fractions. The presence of H lignin in pectin fractions is consistent with the observation that H lignin is deposited in middle lamella and cell wall corners (Donaldson, 2001) and hence co-extracted with pectin. The observation that a major portion of the H lignin signals were found in the 1 M KOH fraction suggests either that this portion of H lignin is directly or indirectly (e.g., via pectin) connected to the KOH-solubilized xylan or xyloglucan in these fractions, or that H lignin is connected through alkaline-labile ester linkages (Balakshin et al., 2011), and thus, extracted in alkaline buffers. Specific linkages between the hemicellulose/pectin/H lignin, however, remain to be determined. H lignin signals in *irx8* appeared to be slightly more prominent than in the WT, possibly due to the lower amount of xylan in the 1 M KOH extract of *irx8* (Figure 7), resulting in an increased lignin to xylan weight ratio. The total amount of H lignin in *irx8*, however, was similar to that of WT as determined by pyMBMS (Figure 4I). Thus, the results do not support a role for GAUT12 in producing a structure required for H lignin deposition.

It is estimated that the chlorite extractions conducted in this study could remove 50–80% of total lignin based on studies of the efficiency of acidic sodium chlorite treatment in the removal of lignin in black spruce, switchgrass, and poplar (Ahlgren and Goring, 1971; Kumar et al., 2013). Therefore, the lignin signals observed in the chlorite- and PC4MKOH extracts by HSQC NMR were likely portions of the residual lignin (20–50%) recovered from each extraction at, or after, the sodium chlorite treatment. This may explain why we did not identify S lignin signals across all fractions, although it is unclear whether S lignin is composed of smaller molecules and depleted during sequential extractions and dialyses. The G lignin in WT was identified mostly in the chlorite- and PC4MKOH extracts (Figures 5A, 6E), indicating that a portion of G lignin is linked to wall polysaccharides via alkaline-resistant linkages, such as benzyl ethers and phenyl glucosides, and that only harsh conditions like acidic sodium chlorite are able to degrade these linkages. In *irx8*, however, the chlorite fraction is nearly depleted of G lignin signals (Figure 5B). Surprisingly, the bulk of the *irx8* G lignin signals, albeit much reduced in amount compared to the WT, was found in the 1 M KOH extract (Figure 6B), a wall fraction released prior to the acidic chlorite treatment, clearly indicating an altered G lignin extractability in the *irx8* mutant. It remains unknown, however, whether there is a portion of G lignin in the *irx8* mutant that does not withstand the acidic chlorite treatment and hence is removed during subsequent dialysis. Overall the results are consistent with the reduced phloroglucinol-HCl staining of *irx8* stem cross-sections (Figure 4B) and the reduced G lignin monomer content identified by pyMBMS (Figure 4I). Our results suggest that in the *irx8* mutant either (i) the G lignin linkages themselves or (ii) the polymers to which the G lignin is connected are partially alkaline-labile, and hence, more easily extracted from the walls in this mutant than in the WT.

### Interdependence of lignin, pectin and xylan in wall biogenesis

The pleiotropic effects of *irx8* on xylan, lignin, and pectin during secondary wall formation confirms that normal wall biogenesis is dependent on an interaction between different cell wall polymers and suggests that there may be a requisite order in which they are deposited *in muro*. Lignification, which creates a relatively rigid and impermeable resin within the polysaccharide network of the wall, has been proposed to occur in two distinct stages. In the primary wall it appears that lignin deposition starts as early as during the formation of the middle lamella at the cell plate, followed by deposition at the cell wall corners—a sequence that has been taken to imply the existence of possible pectin initiation/nucleation sites for primary wall lignin deposition (Donaldson, 2001). Our data demonstrate that Arabidopsis interfascicular fiber tricellular junctions are filled with un-esterified to low-esterified HG, as recognized in a solid triangle shape by anti-HG antibodies CCRC-M38 and JIM5 (Figure 8). This tricellular junction triangle also contains a small amount of high-esterified HG as labeled by JIM7 (Supplemental Figure S9). RG-I backbone, as recognized by CCRC-M14, appears to be present toward the outer layer of this HG triangle, where labeling with this antibody is observed as an empty triangle at the tricellular junction (Figure 8). The lignin in this tricellular junction area was solidly stained for both G and S lignin in *irx8* fibers (Figures 4B,E), suggesting that this process is unlikely to have been affected by the mutation in *GAUT12*. In a second stage of lignification, secondary cell wall lignin is deposited in specific, terminally differentiated cell types (Donaldson, 2001). We showed by colorimetric staining that both G and S lignin are reduced in the *irx8* xylem vessels and fiber cells, apparently due to the reduction in wall thicknesses in these cells. The G lignin in *irx8* is significantly reduced (Figures 4I, 5B), and released in the 1 M KOH extract (Figure 6B) rather than in the chlorite- and PC4MKOH extracts as occurs in the WT (Figures 5A, 6E), suggesting a possible correlation between the xylan reduction and lignin alteration in *irx8*. We also found a significant reduction in expression of four key lignin biosynthetic enzymes (C4H, C3′H, CCoAOMT1, and COMT1) that may lead to reduced lignin precursor production in *irx8* (Figure 4G). These results are consistent with a reduction in total lignin in *irx8* walls, in particular through a reduction in G lignin. Our data support the proposition that lignin formation is down-regulated in a xylan defective mutant, and that reduced *GAUT12* function affects both xylan and lignin deposition.

It has been reported that loss of the primary cellulose synthase subunit CESA3 in the *eli1* mutant is associated with ectopic lignin deposition and increased defense responses (Cano-Delgado et al., 2003), which was interpreted as an effort by the cells to maintain cell wall integrity. By contrast, a decrease in lignin was observed in the *irx8* mutant, and reportedly also in the *irx7* and *irx9* mutants (Petersen et al., 2012). These observations show that loss of matrix polysaccharides causes a reduction in lignin in such *irx* mutants and suggest that matrix polysaccharides provide a different structural function than cellulose during lignification. The reduction of lignin in the *irx8* mutant may be a secondary effect due to the ~60% loss of xylan in *irx8*, since linkages between xylan and lignin have been reported. For example, 4-*O*-methylglucuronoxylan is the major carbohydrate linked to lignin in wood (Yuan et al., 2011) and xylan and lignin are linked via ferulate esters in maize (Grabber et al., 2000). The exact lignin-carbohydrate linkages in the *irx8* mutant are currently under investigation.

### GAUT12 function is required to establish a lamellate structure in the secondary cell wall

We observed interesting lamellate-like patterns of xylan labeling in wild-type fiber cell walls upon immunolabeling with selected xylan-directed antibodies (Figure 8; Supplemental Figure S9). These results suggest that the deposition of different xylan structure may be spatially controlled. LM10 and LM11 bind to xylo-oligomers as small as a disaccharide, while CCRC-M149 binding requires an unsubstituted xylotriose structure, and CCRC-M138 and CCRC-M160 require unsubstituted xylopentaose for recognition (S. Pattathil, U. Avci, and M.G. Hahn, unpublished results). In wild-type fiber cells, LM10 labeling is more intense on the side of the fiber wall adjacent to adjoining cells, and the labeling decreases in intensity toward the lumen side of the wall. LM11 has an overall dotted labeling pattern that covers the entire secondary wall in fiber cells. CCRC-M137 and CCRC-M149 show comparable and relatively even labeling throughout fiber walls. All four antibodies had reduced labeling in *irx8* fiber cell walls due either to a reduced number of epitopes recognized by these antibodies or to reduced thickness of the wall. The fiber cells in *GAUT12*-complemented (*irx8+GAUT12*) plants, however, show a partial restoration of the WT labeling patterns with LM10, LM11, and CCRC-M137. Although *irx8+GAUT12* fibers have thicker walls than those of *irx8*, the lower labeling intensity (i.e., epitope density) with LM10, LM11, and CCRC-M137 in *irx8+GAUT12* fibers resembles that of the *irx8* mutant (Supplemental Figure S9). In contrast, CCRC-M149 labels a thinner wall in *irx8* fibers with similar intensity (i.e., epitope density) in both *irx8* and *irx8+GAUT12* plants compared to WT plants, suggesting that the CCRC-M149-reactive xylan was perhaps indirectly affected by the *irx8* mutation. In other words, the reduction in CCRC-149 labeling in *irx8* fiber walls is likely due to reduced wall thickness and not reduced epitope density.

The most interesting labeling pattern was observed using antibodies CCRC-M138 and CCRC-M160, which showed double-ring labeling patterns in wild-type fiber cell walls (Figure 8; Supplemental Figure S9). The pattern suggests that the middle layer of wild-type fiber secondary walls between the labeled double rings contains xylan (as recognized by CCRC-M149) with fewer regions of unsubstituted xylan as recognized by CCRC-M138. The double-ring still exists in *irx8* fiber cell walls, as can be clearly seen at cell corners, but it has collapsed in *irx8* fiber cell walls due to reduced thickness of the middle layer material of the wall. In addition, the texture of the CCRC-M138 labeling in *irx8* fiber walls is different than in the WT, indicating a re-organized secondary wall with an altered configuration of xylan deposition in the *irx8* mutant. This was also reflected in the altered xylan extraction patterns in *irx8* observed by glycome profiling, revealing that significantly less xylan was extracted in the ammonium oxalate-, sodium carbonate-, and 1 M KOH extracts in the *irx8* mutant walls compared to WT walls (Figure 7). It is unclear why CCRC-M138 labeling is slightly uneven in the *GAUT12*-complemented line (Supplemental Figure S9). A possible explanation is that the 35S promoter drives an inconsistent expression of *GAUT12* leading to the uneven production of corresponding xylan epitopes.

The cell wall immunolabeling patterns in fiber cell cross sections obtained using the six monoclonal antibodies reactive against diverse xylan epitopes indicates that GAUT12 is required for the formation of a middle xylan-containing layer located between an outer (middle lamella-proximal) and an inner (plasma membrane-proximal) xylan layer. These three layers are clearly seen in cross sections of WT fiber cells immunolabeled using antibodies LM11, CCRC-M138 and CCRC-M160. Immunolabeling of fiber cell cross sections of the *irx8* mutant reveals a loss of the middle xylan-containing layer and a collapse of the inner and outer xylan layers onto each other. Since several of the xylan-directed antibodies label the middle layer, as well as the outer and inner xylan layers (e.g., CCRC-M137 and CCRC-M149), it is clear that the middle layer contains xylan. Thus, the lack of the middle layer, and the retention of the outer and inner xylan layers, albeit in a discontinuous pattern in the *irx8* mutants, indicate that GAUT12 is required to produce a WT xylan architecture and that, in the absence of GAUT12, this architecture is either not made or collapses. The current results do not clarify whether the reduction in xylan in the *irx8* mutant is due to the inability of the plant to make a subfraction of xylan that requires GAUT12 for synthesis, or rather, whether the absence of GAUT12 results in an altered xylan architecture that leads to an accumulation of surplus xylan which acts as a negative signal to down-regulate xylan synthesis.

### GAUT12 function

How may the complex phenotype of *irx8* mutants be explained? The reduction in α-1,4-linked GalA in the endopolygalacturonase/pectin methylesterase (EPG/PME)-accessible fraction of *irx8* mutant walls raises the possibility that GAUT12 is involved in the synthesis of an as-yet-to-be-determined HG species. Such a GAUT12-dependent HG may be tightly associated with xylan and, when missing, disrupt xylan biogenesis and/or deposition (Persson et al., 2007). Such GAUT12-dependent pectin and/or xylan may further be a foundation upon which G lignin accumulates.

Recently, Tan et al. (2013) discovered a novel cell wall structure isolated from Arabidopsis suspension cell cultures named ARABINOXYLAN PECTIN ARABINOGALACTAN PROTEIN1 (APAP1), which contains a unique proteoglycan with xylan connected to stretches of RG-I that are flanked by short HG oligomers (Tan et al., 2013). Although we show in the present study that GAUT12 does not appear to have GAUT1-like HG:GalAT activity, i.e., GAUT12 does not catalyze the addition of GalA from UDP-α-GalA onto oligogalacturonide (DP 7–23) acceptors (Figure 9C), a possible function for GAUT12 is the synthesis of such an HG sub-domain using the RG-I sub-domain (oligomer) as a primer for synthesis of an APAP1-like structure in fiber walls. This hypothesis is consistent with the significant decreases in Xyl and GalA in *irx8* stem cell walls (Supplemental Figure S2C) and with the increased immunolabeling of fiber walls with the RG-I backbone-reactive antibody CCRC-M14 (Figure 8). Although there is no obvious change in the wall glycosyl residue composition when *GAUT12* is over-expressed in the WT background (Supplemental Figure S10E), *GAUT12* over-expression may lead to increased production of RG-I/HG sub-domains which may overload the capacity of the xylan biosynthesis machinery, resulting in accumulation of the CCRC-M14 reactive material at the plasma membrane as observed in the WT+*GAUT12* fiber cell walls (Figure 8).

Alternatively, the reduction in the *irx8* mutant of the xylan reducing end sequence [XRES, β-d-Xyl*p*-(1→3)-α-l-Rha*p*-(1→2)-α-d-Gal*p*A-(1→4)-d-Xyl*p*] led to speculations that GAUT12 may catalyze the addition of GalA into the nascent XRES (Peña et al., 2007). However, it remains unknown how XRES synthesis is initiated or whether it acts as a primer or terminator during xylan biosynthesis (York and O'Neill, 2008). *GAUT12* appears not to have any functional homologs in graminaceous monocots (Caffall et al., 2009; Yin et al., 2010). The absence of XRES in grass species examined to date (Kulkarni et al., 2012) would be consistent with a function of GAUT12 in the synthesis of this structure. So far, however, we were not able to demonstrate that GAUT12 adds the GalA into the XRES. Further study is required to identify GAUT12 enzymatic function, as well as its role in the carbohydrate-lignin connection.

In summary, we demonstrate that GAUT12 has a role in anther dehiscence and affects the amount of G lignin and its connectivity to xylan in Arabidopsis. Our work shows that a mutation in a single glycosyltransferase leads to alterations in xylan, pectin, and lignin, thereby providing further evidence for possible associations or connections between different wall polymers. Although the catalytic activity of GAUT12 remains to be determined, we have shown that GAUT12 is not an HG:GalAT with substrate specificities comparable to GAUT1 and that the product of GAUT12 may connect to a structure that contains RG-I and that is required for native xylan architecture in the secondary cell wall.

### Conflict of interest statement

The authors declare that the research was conducted in the absence of any commercial or financial relationships that could be construed as a potential conflict of interest.

## References

[B1] Ahlgren P. A. Goring D. A. I. (1971). Removal of wood components during chlorite delignification of black spruce. Can. J. Chem. 49, 1272–1275 10.1139/V71-207

[B2] Albersheim P. Nevins D. J. English P. D. Karr A. (1967). A method for the analysis of sugars in plant cell-wall polysaccharides by gas-liquid chromatography. Carbohydr. Res. 5, 340–345

[B3] Ariizumi T. Toriyama K. (2011). Genetic regulation of sporopollenin synthesis and pollen exine development. Annu. Rev. Plant Biol. 62, 437–460 10.1146/annurev-arplant-042809-112312 21275644

[B4] Arioli T. Peng L. Betzner A. S. Burn J. Wittke W. Herth W. (1998). Molecular analysis of cellulose biosynthesis in Arabidopsis. Science 279, 717–720 10.1126/science.279.5351.717 9445479

[B5] Atmodjo M. A. Sakuragi Y. Zhu X. Burrell A. J. Mohanty S. S. Atwood J. A. 3rd. (2011). Galacturonosyltransferase (GAUT)1 and GAUT7 are the core of a plant cell wall pectin biosynthetic homogalacturonan:galacturonosyltransferase complex. Proc. Natl. Acad. Sci. U.S.A. 108, 20225–20230 10.1073/pnas.1112816108 22135470 PMC3250160

[B6] Avci U. Pattathil S. Hahn M. G. (2012). Immunological approaches to plant cell wall and biomass characterization: immunolocalization of glycan epitopes. Methods Mol. Biol. 908, 73–82 10.1007/978-1-61779-956-3_7 22843390

[B7] Balakshin M. Capanema E. Gracz H. Chang H. M. Jameel H. (2011). Quantification of lignin-carbohydrate linkages with high-resolution NMR spectroscopy. Planta 233, 1097–1110 10.1007/s00425-011-1359-2 21298285

[B8] Boerjan W. Ralph J. Baucher M. (2003). Lignin biosynthesis. Annu. Rev. Plant Biol. 54, 519–546 10.1146/annurev.arplant.54.031902.134938 14503002

[B9] Bouton S. Leboeuf E. Mouille G. Leydecker M. T. Talbotec J. Granier F. (2002). *QUASIMODO1* encodes a putative membrane-bound glycosyltransferase required for normal pectin synthesis and cell adhesion in Arabidopsis. Plant Cell 14, 2577–2590 10.1105/tpc.004259 12368506 PMC151237

[B10] Bowman J. (1994). Arabidopsis: an Atlas of Morphology and Development. London: Springer, Limited, 2011

[B11] Brown D. M. Goubet F. Wong V. W. Goodacre R. Stephens E. Dupree P. (2007). Comparison of five xylan synthesis mutants reveals new insight into the mechanisms of xylan synthesis. Plant J. 52, 1154–1168 10.1111/j.1365-313X.2007.03307.x 17944810

[B12] Brown D. M. Zeef L. A. Ellis J. Goodacre R. Turner S. R. (2005). Identification of novel genes in Arabidopsis involved in secondary cell wall formation using expression profiling and reverse genetics. Plant Cell 17, 2281–2295 10.1105/tpc.105.031542 15980264 PMC1182489

[B13] Caffall K. H. Pattathil S. Phillips S. E. Hahn M. G. Mohnen D. (2009). *Arabidopsis thaliana* T-DNA mutants implicate *GAUT* genes in the biosynthesis of pectin and xylan in cell walls and seed testa. Mol. Plant 2, 1000–1014 10.1093/mp/ssp062 19825675

[B14] Cano-Delgado A. Penfield S. Smith C. Catley M. Bevan M. (2003). Reduced cellulose synthesis invokes lignification and defense responses in *Arabidopsis thaliana*. Plant J. 34, 351–362 10.1046/j.1365-313X.2003.01729.x 12713541

[B15] Chapple C. C. Vogt T. Ellis B. E. Somerville C. R. (1992). An Arabidopsis mutant defective in the general phenylpropanoid pathway. Plant Cell 4, 1413–1424 10.1105/tpc.4.11.1413 1477555 PMC160228

[B16] Clough S. J. Bent A. F. (1998). Floral dip: a simplified method for *Agrobacterium*-mediated transformation of *Arabidopsis thaliana*. Plant J. 16, 735–743 10.1046/j.1365-313x.1998.00343.x 10069079

[B17] Dardelle F. Lehner A. Ramdani Y. Bardor M. Lerouge P. Driouich A. (2010). Biochemical and immunocytological characterizations of Arabidopsis pollen tube cell wall. Plant Physiol. 153, 1563–1576 10.1104/pp.110.158881 20547702 PMC2923879

[B18] Demartini J. D. Pattathil S. Avci U. Szekalski K. Mazumder K. Hahn M. G. (2011). Application of monoclonal antibodies to investigate plant cell wall deconstruction for biofuels production. Energy Environ. Sci. 4, 4332–4339 10.1039/c1ee02112e

[B19] Doelling J. H. Pikaard C. S. (1993). Transient expression in *Arabidopsis thaliana* protoplasts derived from rapidly established cell-suspension cultures. Plant Cell Rep. 12, 241–244 10.1007/BF00237127 24197149

[B20] Donaldson L. A. (2001). Lignification and lignin topochemistry—an ultrastructural view. Phytochemistry 57, 859–873 10.1016/S0031-9422(01)00049-8 11423137

[B21] Donaldson L. A. Knox J. P. (2012). Localization of cell wall polysaccharides in normal and compression wood of radiata pine: relationships with lignification and microfibril orientation. Plant Physiol. 158, 642–653 10.1104/pp.111.184036 22147521 PMC3271756

[B22] Doong R. L. Mohnen D. (1998). Solubilization and characterization of a galacturonosyltransferase that synthesizes the pectic polysaccharide homogalacturonan. Plant J. 13, 363–374 10.1046/j.1365-313X.1998.00042.x

[B23] Evans R. J. Milne T. A. (1987). Molecular characterization of the pyrolysis of biomass.1. fundamentals. Energy Fuels 1, 123–137 10.1021/Ef00002a001

[B24] Grabber J. H. Ralph J. Hatfield R. D. (2000). Cross-linking of maize walls by ferulate dimerization and incorporation into lignin. J. Agric. Food Chem. 48, 6106–6113 10.1021/jf0006978 11312783

[B25] Guo D. Chen F. Inoue K. Blount J. W. Dixon R. A. (2001). Downregulation of caffeic acid 3-*O*-methyltransferase and caffeoyl CoA 3-*O*-methyltransferase in transgenic alfalfa. impacts on lignin structure and implications for the biosynthesis of G and S lignin. Plant Cell 13, 73–88 10.1105/tpc.13.1.73 11158530 PMC102215

[B26] Jeffries T. W. (1990). Biodegradation of lignin-carbohydrate complexes. Biodegradation 1, 163–176 10.1007/bf00058834

[B27] Jiang N. Pu Y. Q. Samuel R. Ragauskas A. J. (2009). Perdeuterated pyridinium molten salt (ionic liquid) for direct dissolution and NMR analysis of plant cell walls. Green Chem. 11, 1762–1766 10.1039/b913609f

[B28] Jung K. W. Oh S. I. Kim Y. Y. Yoo K. S. Cui M. H. Shin J. S. (2008). Arabidopsis histidine-containing phosphotransfer factor 4 (AHP4) negatively regulates secondary wall thickening of the anther endothecium during flowering. Mol. Cells 25, 294–300 18413999

[B29] Kim H. Ralph J. (2010). Solution-state 2D NMR of ball-milled plant cell wall gels in DMSO-*d*_6_/pyridine-*d*_5_. Org. Biomol. Chem. 8, 576–591 10.1039/b916070a 20090974 PMC4070321

[B30] Ko J. H. Kim W. C. Han K. H. (2009). Ectopic expression of MYB46 identifies transcriptional regulatory genes involved in secondary wall biosynthesis in Arabidopsis. Plant J. 60, 649–665 10.1111/j.1365-313X.2009.03989.x 19674407

[B31] Kong Y. Zhou G. Avci U. Gu X. Jones C. Yin Y. (2009). Two poplar glycosyltransferase genes, *PdGATL1.1* and *PdGATL1.2*, are functional orthologs to *PARVUS/AtGATL1* in *Arabidopsis*. Mol. Plant 2, 1040–1050 10.1093/mp/ssp068 19825678

[B32] Kulkarni A. R. Peña M. J. Avci U. Mazumder K. Urbanowicz B. R. Pattathil S. (2012). The ability of land plants to synthesize glucuronoxylans predates the evolution of tracheophytes. Glycobiology 22, 439–451 10.1093/glycob/cwr117 22048859

[B33] Kumar R. Hu F. Hubbell C. A. Ragauskas A. J. Wyman C. E. (2013). Comparison of laboratory delignification methods, their selectivity, and impacts on physiochemical characteristics of cellulosic biomass. Bioresour. Technol. 130, 372–381 10.1016/j.biortech.2012.12.028 23313683

[B34] Lao N. T. Long D. Kiang S. Coupland G. Shoue D. A. Carpita N. C. (2003). Mutation of a family 8 glycosyltransferase gene alters cell wall carbohydrate composition and causes a humidity-sensitive semi-sterile dwarf phenotype in *Arabidopsis*. Plant Mol. Biol. 53, 647–661 10.1023/B:PLAN.0000019074.60542.6c 15010604

[B35] Leboeuf E. Guillon F. Thoiron S. Lahaye M. (2005). Biochemical and immunohistochemical analysis of pectic polysaccharides in the cell walls of *Arabidopsis* mutant *QUASIMODO 1* suspension-cultured cells: implications for cell adhesion. J. Exp. Bot. 56, 3171–3182 10.1093/jxb/eri314 16263905

[B36] Lee C. O'Neill M. A. Tsumuraya Y. Darvill A. G. Ye Z. H. (2007a). The *irregular xylem9* mutant is deficient in xylan xylosyltransferase activity. Plant Cell Physiol. 48, 1624–1634 10.1093/pcp/pcm135 17938130

[B37] Lee C. Zhong R. Richardson E. A. Himmelsbach D. S. McPhail B. T. Ye Z. H. (2007b). The *PARVUS* gene is expressed in cells undergoing secondary wall thickening and is essential for glucuronoxylan biosynthesis. Plant Cell Physiol. 48, 1659–1672 10.1093/pcp/pcm155 17991630

[B38] Lewis N. G. Yamamoto E. (1990). Lignin: occurrence, biogenesis and biodegradation. Annu. Rev. Plant Physiol. Plant Mol. Biol. 41, 455–496 10.1146/annurev.pp.41.060190.002323 11543592

[B39] Livak K. J. Schmittgen T. D. (2001). Analysis of relative gene expression data using real-time quantitative PCR and the 2^-ΔΔCT^ method. Methods 25, 402–408 10.1006/meth.2001.1262 11846609

[B40] McCarthy R. L. Zhong R. Ye Z. H. (2009). MYB83 is a direct target of SND1 and acts redundantly with MYB46 in the regulation of secondary cell wall biosynthesis in Arabidopsis. Plant Cell Physiol. 50, 1950–1964 10.1093/pcp/pcp139 19808805

[B41] McCartney L. Marcus S. E. Knox J. P. (2005). Monoclonal antibodies to plant cell wall xylans and arabinoxylans. J. Histochem. Cytochem. 53, 543–546 10.1369/jhc.4B6578.2005 15805428

[B42] Mitsuda N. Seki M. Shinozaki K. Ohme-Takagi M. (2005). The NAC transcription factors NST1 and NST2 of Arabidopsis regulate secondary wall thickenings and are required for anther dehiscence. Plant Cell 17, 2993–3006 10.1105/tpc.105.036004 16214898 PMC1276025

[B43] Orfila C. Sorensen S. O. Harholt J. Geshi N. Crombie H. Truong H. N. (2005). *QUASIMODO1* is expressed in vascular tissue of *Arabidopsis thaliana* inflorescence stems, and affects homogalacturonan and xylan biosynthesis. Planta 222, 613–622 10.1007/s00425-005-0008-z 16059719

[B44] Pattathil S. Avci U. Baldwin D. Swennes A. G. McGill J. A. Popper Z. (2010). A comprehensive toolkit of plant cell wall glycan-directed monoclonal antibodies. Plant Physiol. 153, 514–525 10.1104/pp.109.151985 20363856 PMC2879786

[B45] Pattathil S. Avci U. Miller J. S. Hahn M. G. (2012). Immunological approaches to plant cell wall and biomass characterization: glycome profiling. Methods Mol. Biol. 908, 61–72 10.1007/978-1-61779-956-3_6 22843389

[B46] Pattathil S. Harper A. D. Bar-Peled M. (2005). Biosynthesis of UDP-xylose: characterization of membrane-bound *AtUxs2*. Planta 221, 538–548 10.1007/s00425-004-1471-7 15655675

[B47] Pedrioli P. G. Eng J. K. Hubley R. Vogelzang M. Deutsch E. W. Raught B. (2004). A common open representation of mass spectrometry data and its application to proteomics research. Nat. Biotechnol. 22, 1459–1466 10.1038/nbt1031 15529173

[B48] Peña M. J. Zhong R. Zhou G. K. Richardson E. A. O'neill M. A. Darvill A. G. (2007). Arabidopsis *irregular xylem8* and *irregular xylem9*: implications for the complexity of glucuronoxylan biosynthesis. Plant Cell 19, 549–563 10.1105/tpc.106.049320 17322407 PMC1867335

[B49] Persson S. Caffall K. H. Freshour G. Hilley M. T. Bauer S. Poindexter P. (2007). The *Arabidopsis irregular xylem8* mutant is deficient in glucuronoxylan and homogalacturonan, which are essential for secondary cell wall integrity. Plant Cell 19, 237–255 10.1105/tpc.106.047720 17237350 PMC1820957

[B50] Persson S. Wei H. Milne J. Page G. P. Somerville C. R. (2005). Identification of genes required for cellulose synthesis by regression analysis of public microarray data sets. Proc. Natl. Acad. Sci. U.S.A. 102, 8633–8638 10.1073/pnas.0503392102 15932943 PMC1142401

[B51] Petersen P. D. Lau J. Ebert B. Yang F. Verhertbruggen Y. Kim J. S. (2012). Engineering of plants with improved properties as biofuels feedstocks by vessel-specific complementation of xylan biosynthesis mutants. Biotechnol. Biofuels 5:84 10.1186/1754-6834-5-84 23181474 PMC3537538

[B52] Raes J. Rohde A. Christensen J. H. Van De Peer Y. Boerjan W. (2003). Genome-wide characterization of the lignification toolbox in Arabidopsis. Plant Physiol. 133, 1051–1071 10.1104/pp.103.026484 14612585 PMC523881

[B53] Sangha A. K. Parks J. M. Standaert R. F. Ziebell A. Davis M. Smith J. C. (2012). Radical coupling reactions in lignin synthesis: a density functional theory study. J. Phys. Chem. B 116, 4760–4768 10.1021/Jp2122449 22475051

[B54] Scheller H. V. Ulvskov P. (2010). Hemicelluloses. Annu. Rev. Plant Biol. 61, 263–289 10.1146/annurev-arplant-042809-112315 20192742

[B55] Schilmiller A. L. Stout J. Weng J. K. Humphreys J. Ruegger M. O. Chapple C. (2009). Mutations in the *cinnamate 4-hydroxylase* gene impact metabolism, growth and development in Arabidopsis. Plant J. 60, 771–782 10.1111/j.1365-313X.2009.03996.x 19682296

[B56] Shao M. Zheng H. Hu Y. Liu D. Jang J. C. Ma H. (2004). The *GAOLAOZHUANGREN1* gene encodes a putative glycosyltransferase that is critical for normal development and carbohydrate metabolism. Plant Cell Physiol. 45, 1453–1460 10.1093/pcp/pch168 15564529

[B57] Steiner-Lange S. Unte U. S. Eckstein L. Yang C. Wilson Z. A. Schmelzer E. (2003). Disruption of *Arabidopsis thaliana MYB26* results in male sterility due to non-dehiscent anthers. Plant J. 34, 519–528 10.1046/j.1365-313X.2003.01745.x 12753590

[B58] Sterling J. D. Atmodjo M. A. Inwood S. E. Kumar Kolli V. S. Quigley H. F. Hahn M. G. (2006). Functional identification of an Arabidopsis pectin biosynthetic homogalacturonan galacturonosyltransferase. Proc. Natl. Acad. Sci. U.S.A. 103, 5236–5241 10.1073/pnas.0600120103 16540543 PMC1458824

[B59] Sterling J. D. Lemons J. A. Forkner I. F. Mohnen D. (2005). Development of a filter assay for measuring homogalacturonan: α-(1,4)-galacturonosyltransferase activity. Anal. Biochem. 343, 231–236 10.1016/j.ab.2005.05.037 16005842

[B60] Sykes R. Kodrzycki B. Tuskan G. Foutz K. Davis M. (2008). Within tree variability of lignin composition in Populus. Wood Sci. Technol. 42, 649–661 10.1007/s00226-008-0199-0

[B61] Tan L. Eberhard S. Pattathil S. Warder C. Glushka J. Yuan C. (2013). An *Arabidopsis* cell wall proteoglycan consists of pectin and arabinoxylan covalently linked to an arabinogalactan protein. Plant Cell 25, 270–287 10.1105/tpc.112.107334 23371948 PMC3584541

[B62] Taylor N. G. Howells R. M. Huttly A. K. Vickers K. Turner S. R. (2003). Interactions among three distinct CesA proteins essential for cellulose synthesis. Proc. Natl. Acad. Sci. U.S.A. 100, 1450–1455 10.1073/pnas.0337628100 12538856 PMC298793

[B63] Thevenin J. Pollet B. Letarnec B. Saulnier L. Gissot L. Maia-Grondard A. (2011). The simultaneous repression of CCR and CAD, two enzymes of the lignin biosynthetic pathway, results in sterility and dwarfism in *Arabidopsis thaliana*. Mol. Plant 4, 70–82 10.1093/mp/ssq045 20829305

[B64] Wang Y. Zhang W. Z. Song L. F. Zou J. J. Su Z. Wu W. H. (2008). Transcriptome analyses show changes in gene expression to accompany pollen germination and tube growth in Arabidopsis. Plant Physiol. 148, 1201–1211 10.1104/pp.108.126375 18775970 PMC2577266

[B65] Weatherly D. B. Atwood J. A. 3rd. Minning T. A. Cavola C. Tarleton R. L. Orlando R. (2005). A Heuristic method for assigning a false-discovery rate for protein identifications from Mascot database search results. Mol. Cell. Proteomics 4, 762–772 10.1074/mcp.M400215-MCP200 15703444

[B66] Weng J. K. Mo H. Chapple C. (2010). Over-expression of F5H in COMT-deficient Arabidopsis leads to enrichment of an unusual lignin and disruption of pollen wall formation. Plant J. 64, 898–911 10.1111/j.1365-313X.2010.04391.x 21143672

[B67] Wilson Z. A. Song J. Taylor B. Yang C. (2011). The final split: the regulation of anther dehiscence. J. Exp. Bot. 62, 1633–1649 10.1093/jxb/err014 21325605

[B68] Yamaguchi M. Goue N. Igarashi H. Ohtani M. Nakano Y. Mortimer J. C. (2010). VASCULAR-RELATED NAC-DOMAIN6 and VASCULAR-RELATED NAC-DOMAIN7 effectively induce transdifferentiation into xylem vessel elements under control of an induction system. Plant Physiol. 153, 906–914 10.1104/pp.110.154013 20488898 PMC2899931

[B69] Yang C. Xu Z. Song J. Conner K. Vizcay Barrena G. Wilson Z. A. (2007). *Arabidopsis MYB26/MALE STERILE35* regulates secondary thickening in the endothecium and is essential for anther dehiscence. Plant Cell 19, 534–548 10.1105/tpc.106.046391 17329564 PMC1867336

[B70] Yin Y. Chen H. Hahn M. G. Mohnen D. Xu Y. (2010). Evolution and function of the plant cell wall synthesis-related glycosyltransferase family 8. Plant Physiol. 153, 1729–1746 10.1104/pp.110.154229 20522722 PMC2923890

[B71] York W. S. O'Neill M. A. (2008). Biochemical control of xylan biosynthesis—which end is up? Curr. Opin. Plant Biol. 11, 258–265 10.1016/j.pbi.2008.02.007 18374624

[B72] Yuan T. Q. Sun S. N. Xu F. Sun R. C. (2011). Characterization of lignin structures and lignin-carbohydrate complex (LCC) linkages by quantitative ^13^C and 2D HSQC NMR spectroscopy. J. Agric. Food Chem. 59, 10604–10614 10.1021/jf2031549 21879769

[B73] Zhong R. Peña M. J. Zhou G. K. Nairn C. J. Wood-Jones A. Richardson E. A. (2005). *Arabidopsis Fragile Fiber8*, which encodes a putative glucuronyltransferase, is essential for normal secondary wall synthesis. Plant Cell 17, 3390–3408 10.1105/tpc.105.035501 16272433 PMC1315377

[B74] Zou J. Song L. Zhang W. Wang Y. Ruan S. Wu W. H. (2009). Comparative proteomic analysis of Arabidopsis mature pollen and germinated pollen. J. Integr. Plant Biol. 51, 438–455 10.1111/j.1744-7909.2009.00823.x 19508356

