# Supplementary material for: Loss of Arabidopsis GAUT12/IRX8 causes anther indehiscence and leads to reduced G lignin associated with altered matrix polysaccharide deposition
Source: Front Plant Sci. 2014 Jul 28;5:357. doi: 10.3389/fpls.2014.00357 (PMC4112939; doi:10.3389/fpls.2014.00357)
Supplement: Supplementary file 1 [file DataSheet1.PDF]

Loss of Arabidopsis *GAUT12/IRX8* causes anther indehiscence and leads to reduced G lignin associated with altered matrix polysaccharide deposition

Zhangying Hao, Utku Avci, Li Tan, Xiang Zhu, John Glushka, Sivakumar Pattathil, Stefan Eberhard, Tipton Sholes, Grace Rothstein, Wolfgang Lukowitz, Ron Orlando, Michael G. Hahn, Debra Mohnen

Supplementary Data

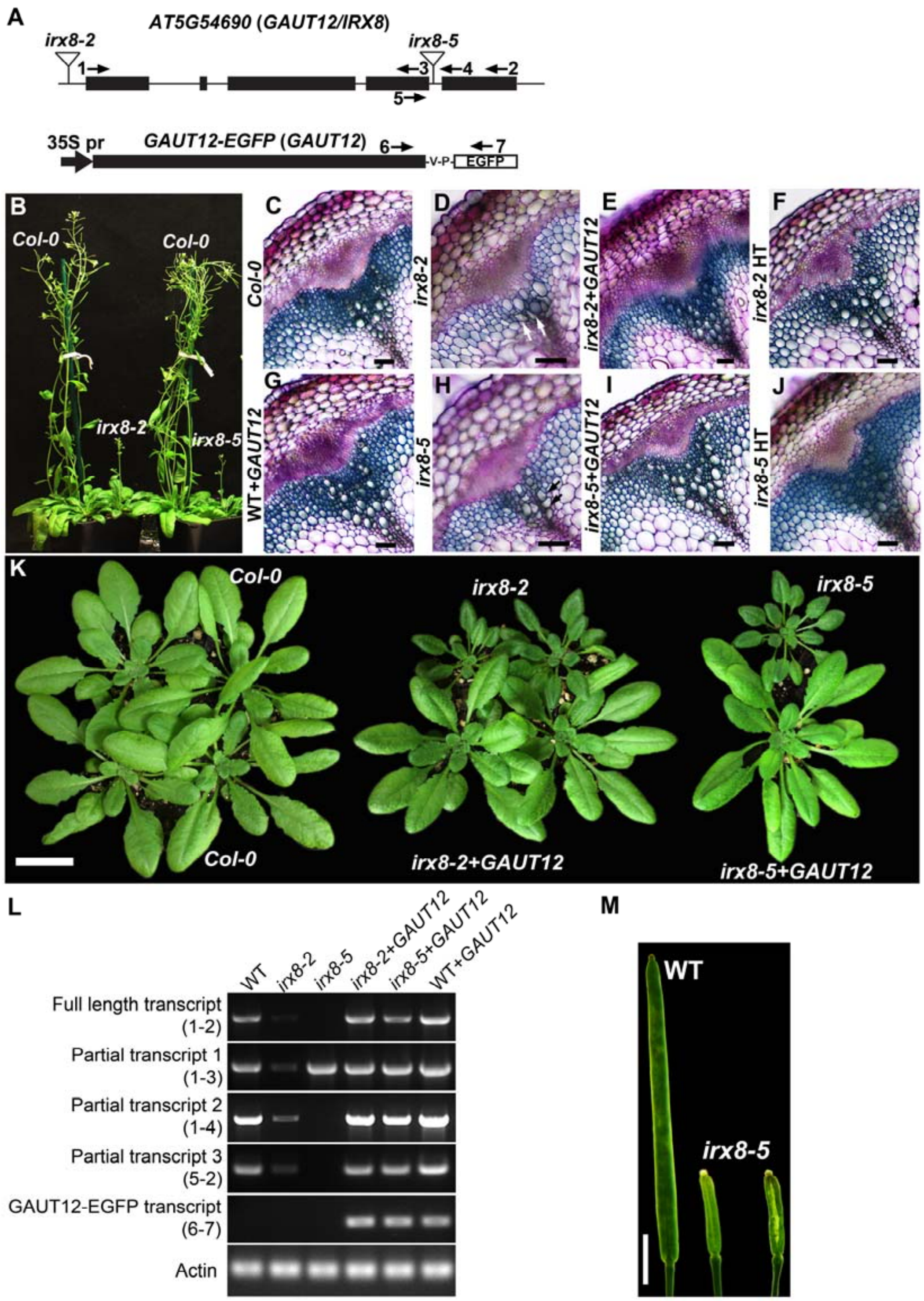

Supplemental Figure S1. The *irx8* mutant alleles are complemented by a *GAUT12-EGFP* construct.

(A) Relative position of T-DNA insertions in *irx8-2* and *irx8-5* and position of primers used to measure transcript levels as indicated by arrows (upper panel). The *GAUT12-EGFP* (*GAUT12*)

construct is driven by the CaMV 35S promoter and contains the full GAUT12 coding sequence followed by enhanced GFP linked via a valine-proline (-V-P-) linker (lower panel).

(B) Seven-week-old *irx8-2* and *irx8-5* mutants have similar dwarf phenotypes. *Col-0*, wild type *Columbia-0*.

(C) to **(J)** Toluidine blue-O stained free-hand transverse sections of WT **(C)**, *irx8-2* **(D)**, *irx8-2* + *GAUT12* **(E)**, *irx8-2* heterozygote (HT, **F**), WT+*GAUT12* **(G)**, *irx8-5* **(H)**, *irx8-5* + *GAUT12* **(I)**, and *irx8-5* HT **(J)**. Arrows in **(D)** indicate collapsed xylem vessels due to reduced wall thickness, which was complemented by the *GAUT12-EGFP* construct. Bar = 50 μm.

(K) Constitutive expression of the *GAUT12-EGFP* construct in both *irx8-2* and *irx8-5* homozygote mutants rescued the rosette leaf size of 5-week-old mutants to wild type levels.

(L) Full length *GAUT12* transcript (by primer 1 and 2 indicated in A) was absent in *irx8-5* but reduced in *irx8-2*, while a partial N-terminal transcript (by primer 1 and 3) was detected in *irx8-5*. Partial transcript 2 (by primer 1 and 4) and 3 (by primer 5 and 2) both spanning the T-DNA site were absent in *irx8-5*. The *GAUT12-EGFP* expression was only detected in the transgenic plants (by primers 6 and 7). Total RNA was harvested from bottom stems (1 inch from soil) of the corresponding plants.

(M) Siliques from WT and *irx8-5* plants. The *irx8-5* silique is smaller and contains almost no seeds. Bar = 2 mm.

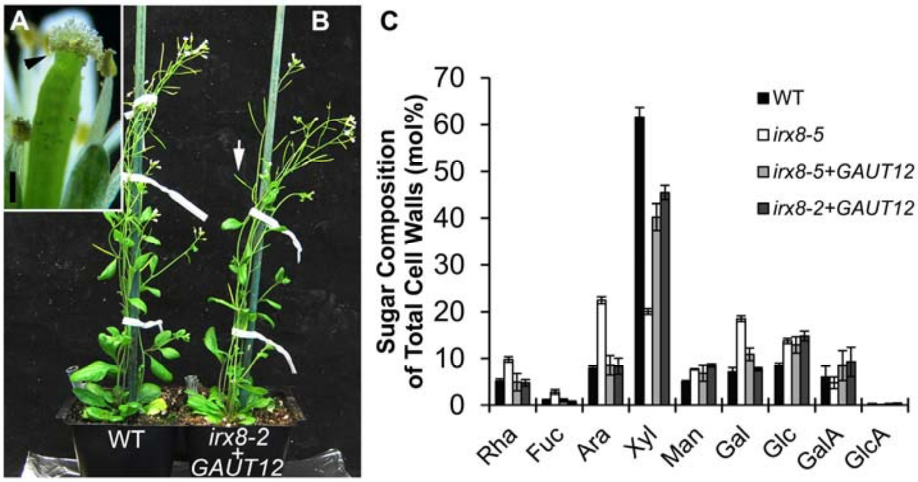

Supplemental Figure S2. The *irx8* indehiscent anther phenotype complemented by *GAUT12-EGFP* (*GAUT12*) and cell wall analyses of WT, *irx8*, and *irx8*-complemented plants.

(A) *irx8-5+GAUT12* flower, the black arrowhead indicates pollen shed onto stigma. Bar = 250 μm.

(B) *irx8-2+GAUT12* plant restored the plant stature to WT-like. White arrow indicates formation of siliques.

(C) Sugar composition of total cell walls (AIR) from 7-week-old plant stems. Neutral sugar composition was determined by gas chromatography-mass spectrometry of alditol acetates. Uronic acids were analyzed by DIONEX. Value = mol% of individual sugar ± standard error.

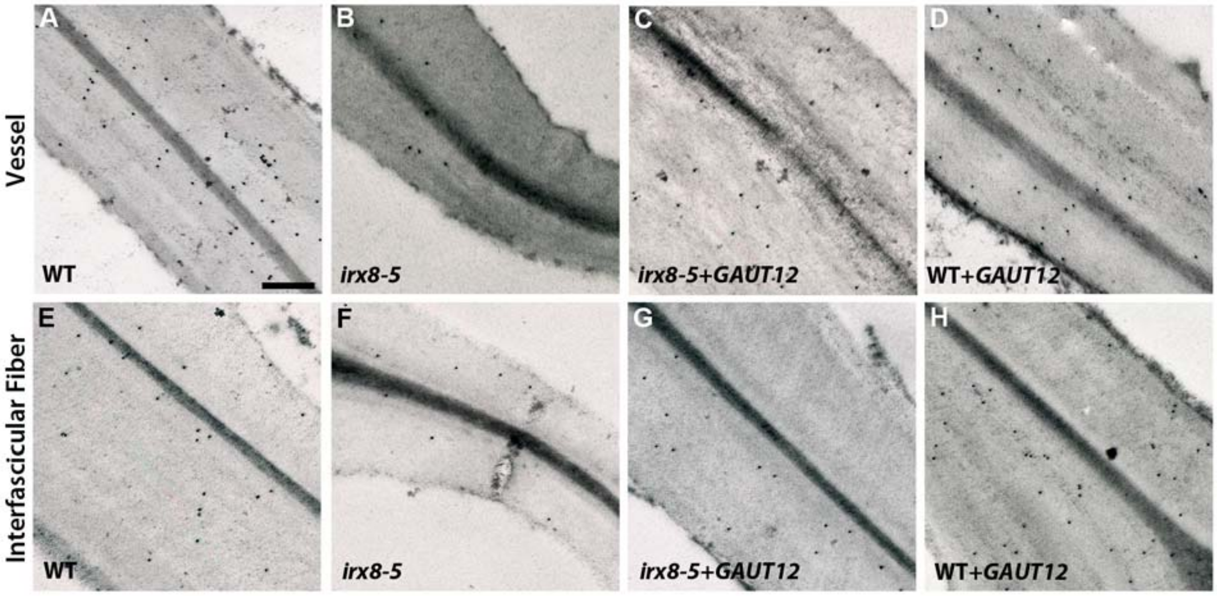

Supplemental Figure S3. TEM of LM10 immunogold-labeled xylem vessel and interfascicular fiber cell walls of WT, *irx8*, *irx8-5+GAUT12* and WT+*GAUT12*.

Xylem vessel cell walls labeled with the anti-xylan antibody LM10 in WT **(A)**, *irx8-5* **(B)**, *irx8+GAUT12* **(C)**, and WT+*GAUT12* **(D)**.

Interfascicular fiber cell walls labeled with LM10 in WT **(E)**, *irx8-5* **(F)**, *irx8+GAUT12* **(G)**, and WT+*GAUT12* **(H)**. Bar = 0.3 μm.

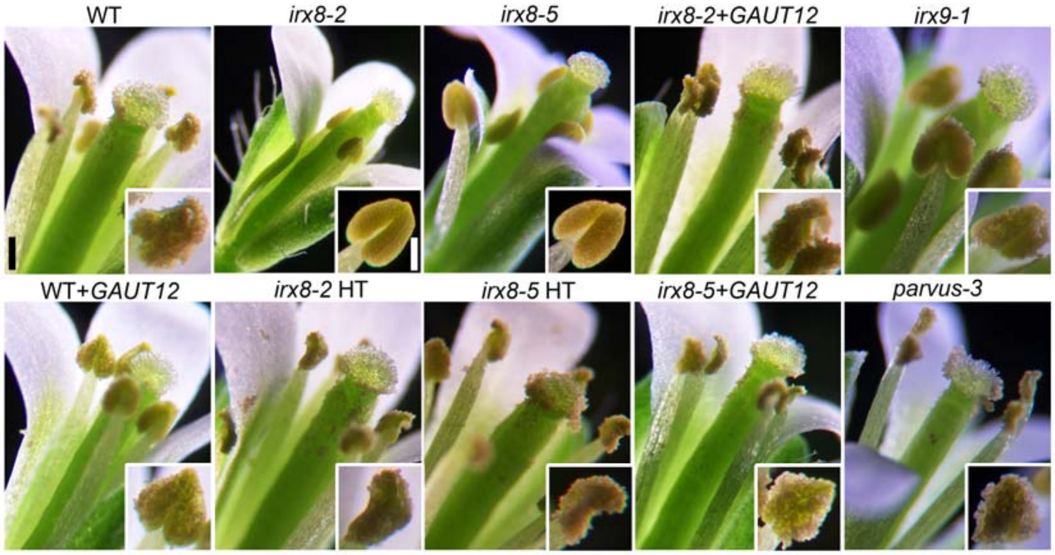

Supplemental Figure S4. Light microscope images of anther and pistil in open flowers of WT, *irx8-2*, *irx8-5*, *irx8-2* and *irx8-5* heterozygote (HT), *irx8-2+GAUT12*, *irx8-5+GAUT12*, WT+*GAUT12*, *irx9-1*, and *parvus-3*.

Both *irx8-5* and *irx8-2* have short anther filaments and indehiscent anthers with no pollen release. The insets show close-up images of an anther from the corresponding flowers. Bar = 250 µm for flowers and = 100 µm for inset anthers.

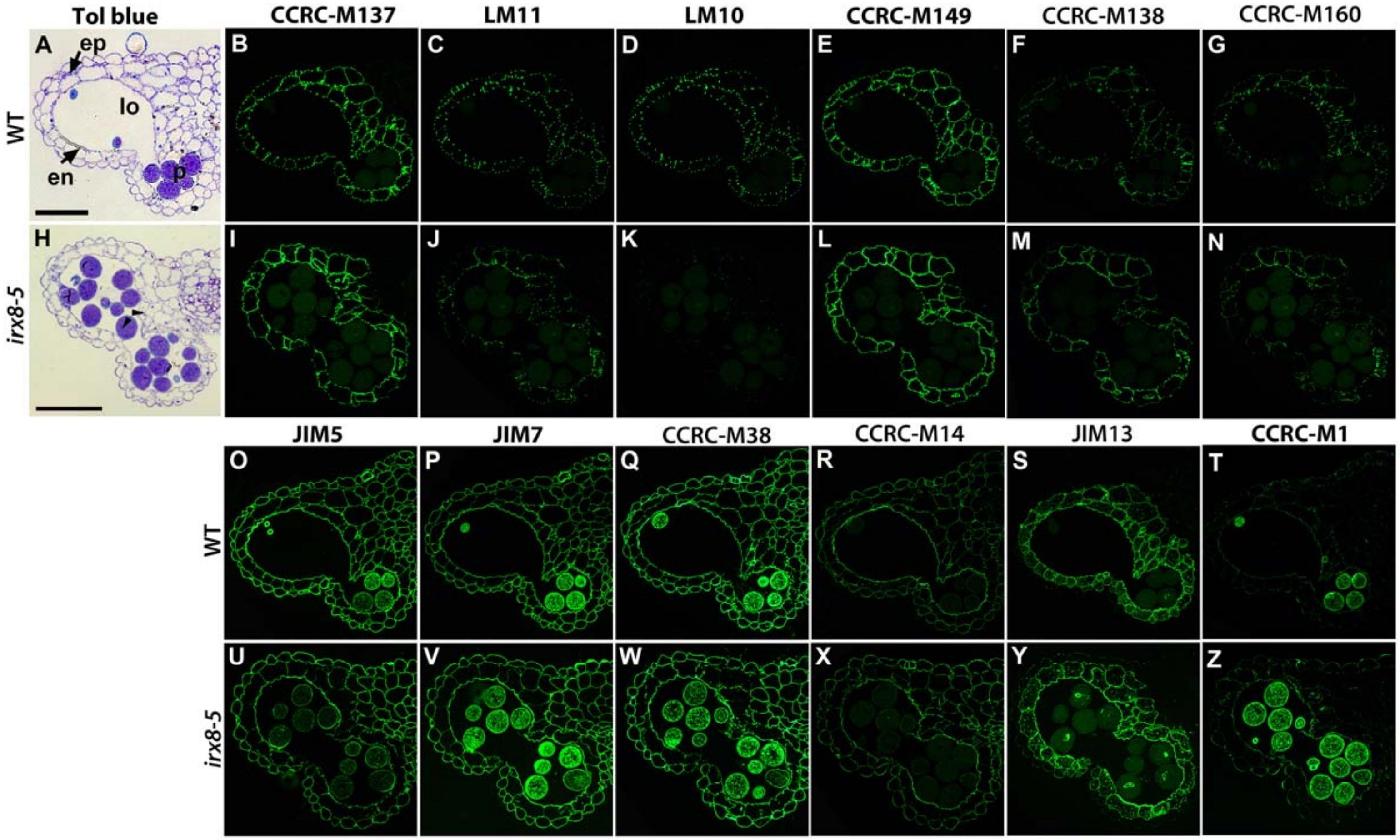
Tol blue
CCRC-M137
LM11
LM10
CCRC-M149
CCRC-M138
CCRC-M160
WT
irx8-5
ep
lo
en
p
JIM5
JIM7
CCRC-M38
CCRC-M14
JIM13
CCRC-M1
A
B
C
D
E
F
G
H
I
J
K
L
M
N
O
P
Q
R
S
T
U
V
W
X
Y
Z

Supplemental Figure S5. Immunofluorescent labeling of transverse-sections of LR White-embedded *irx8* and wild type (WT) anthers in open flowers.

WT **(A)** and *irx8-5* **(H)** anther sections (250-nm-thick) were stained with toluidine blue, arrowheads point to septum breakage. ep, epidermis; en, endothecium; p, pollen; lo, locule. WT and *irx8-5* anthers labeled with xylan-directed antibodies CCRC-M137 (**B** and **I**), LM11 (**C** and **J**), LM10 (**D** and **K**), CCRC-M149 (**E** and **L**), CCRC-M138 (**F** and **M**), and CCRC-M160 (**G** and **N**); pectin-directed antibodies JIM5 (**O** and **U**), JIM7 (**P** and **V**), CCRC-M38 (**Q** and **W**), and CCRC-M14 (**R** and **X**); AGP-directed antibody JIM13 (**S** and **Y**); and fucosylated xyloglucan-directed antibody CCRC-M1 (**T** and **Z**); Bar = 50 μm. CCRC-M38 recognizes un-esterified HG, CCRC-M14 recognizes RG-I backbone, JIM5 recognizes low-esterified HG, JIM7 recognizes high-esterified HG, JIM13 recognizes AGP, LM10 (Xylan-6) binds to low-substituted xylan, LM11 (Xylan-6) binds to both low- and high-substituted xylan, CCRC-M149 (Xylan-7) binds strongly to xylotriose and xylopentaose, CCRC-M138 (Xylan-6) and CCRC-M160 (Xylan-7) bind to xylopentaose. The xylan epitope recognized by CCRC-M137 (xylan-7) remains undefined (Pattathil et al., 2010; Pattathil et al., 2012).

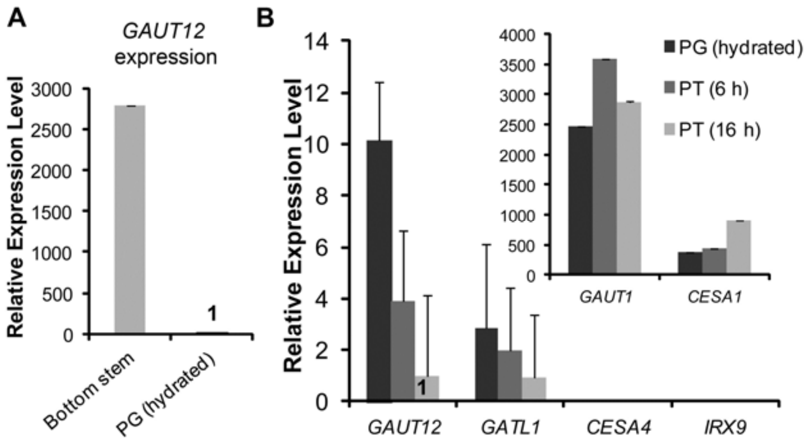

Supplemental Figure S6. *GAUT12* transcript expression in pollen grains (PG) and pollen tubes (PT) measured by qPCR.

(A) *GAUT12* transcript expression in Arabidopsis bottom stems and hydrated pollen grains (*GAUT12* expression in PG was set as 1).

(B) *GAUT12* transcript expression in hydrated pollen grains (0.5 h) and *in vitro* grown pollen tubes (*GAUT12* expression in 16 h PT was set as 1). *CESA4* and *IRX9* showed no expression in either pollen grains or growing pollen tubes. Values = average relative expression level ± standard deviation of three independent experiments.

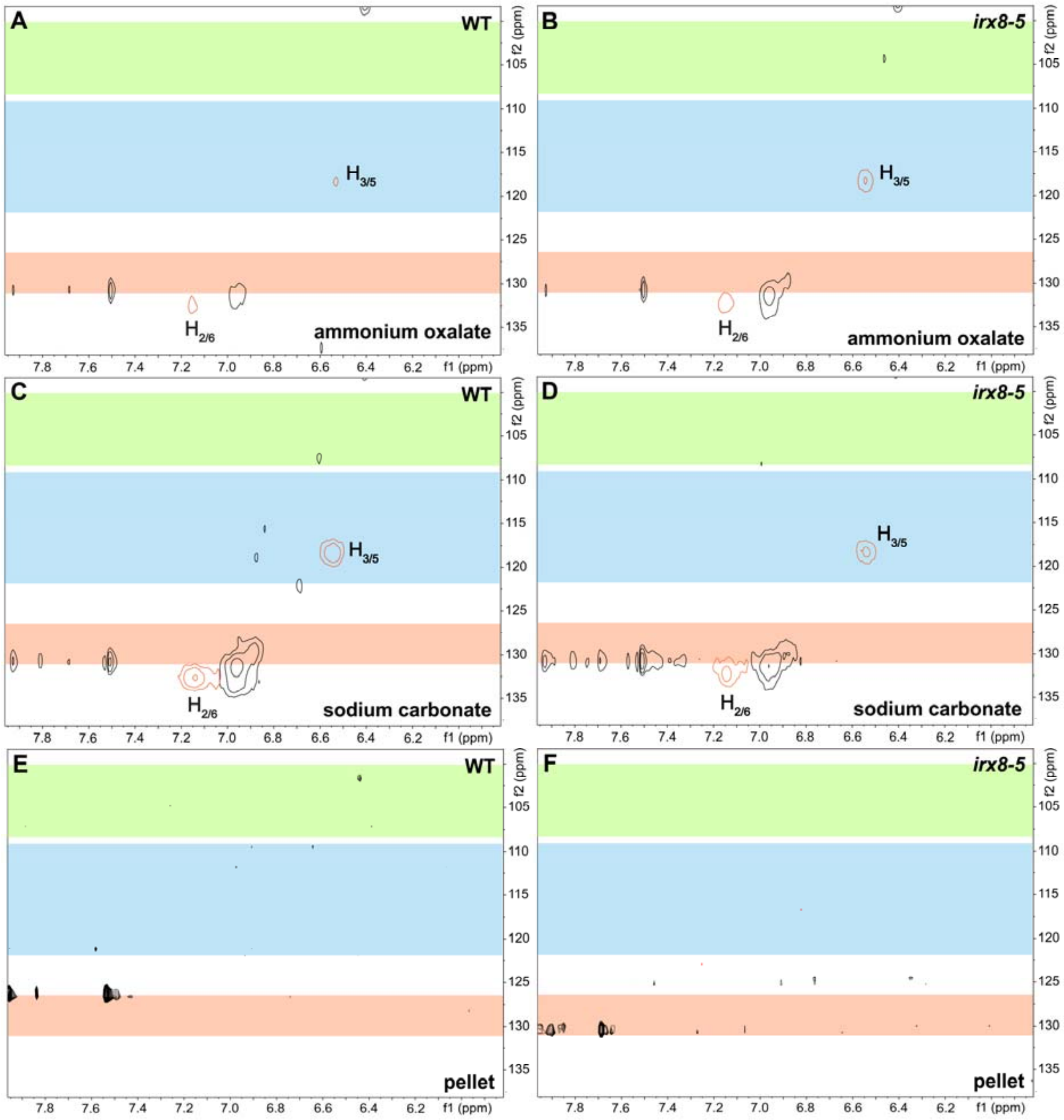

Supplemental Figure S7. Aromatic regions of 2D ^{13}C-^{1}H Heteronuclear Single-Quantum Correlation (HSQC) NMR spectra of ammonium oxalate-, sodium carbonate wall extracts, and residual pellets of WT and the *irx8-5* mutant.

WT **(A)** and *irx8-5* **(B)** ammonium oxalate extracts.

WT **(C)** and *irx8-5* (**D**) sodium carbonate extracts.

WT **(E)** and *irx8-5* **(F)** residual pellets. The signals of H, S, and G lignin monomers are as labeled.

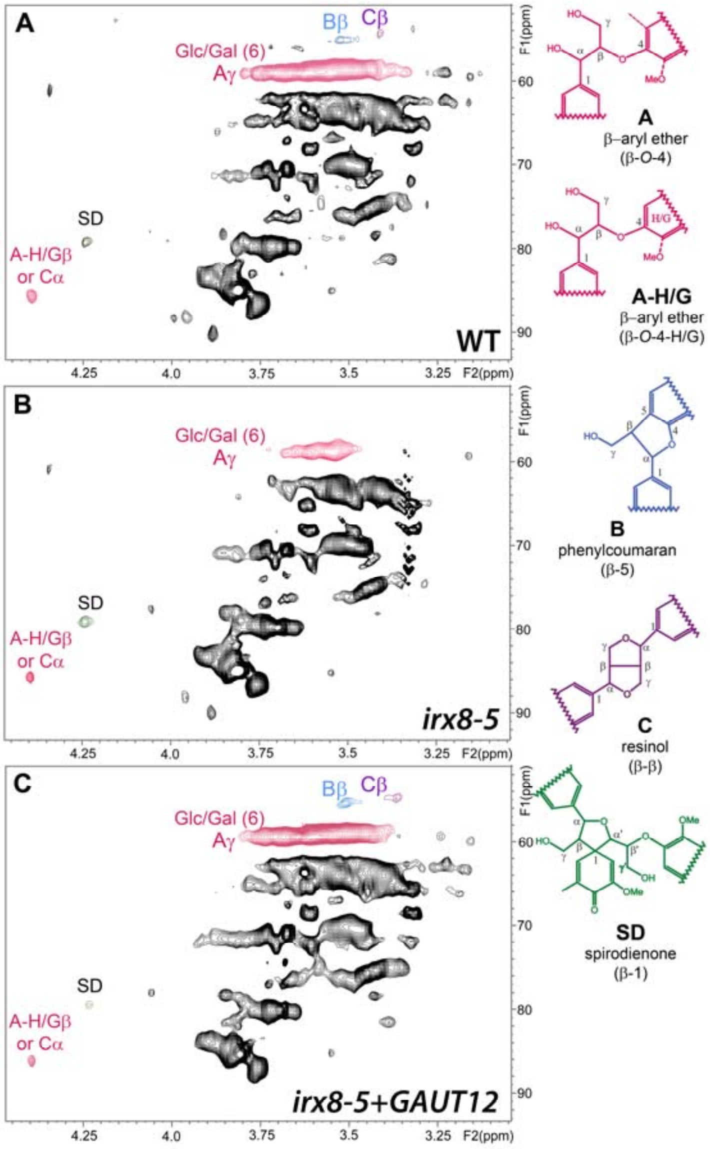

Supplemental Figure S8. Aliphatic sidechain region of 2D ^{13}C-^{1}H Heteronuclear Single-Quantum Correlation (HSQC) spectra of chlorite extracts prepared from WT **(A)**, *irx8-5* **(B)**, and *irx8-5*+*GAUT12* **(C)** stem alcohol insoluble residues (AIR).

Cross peaks representing lignin aliphatic side chains are labeled. Major changes in the *irx8* mutant from the WT were colored according to the lignin structures provided on right. There is a reduction of Glc/Gal (6)/Aγ signal as well as a complete loss of Bβ and Cβ signals in *irx8*, which are recovered in *irx8*+*GAUT12*. Glc/Gal(6) refers to the carbon 6 on glucose or galactose.

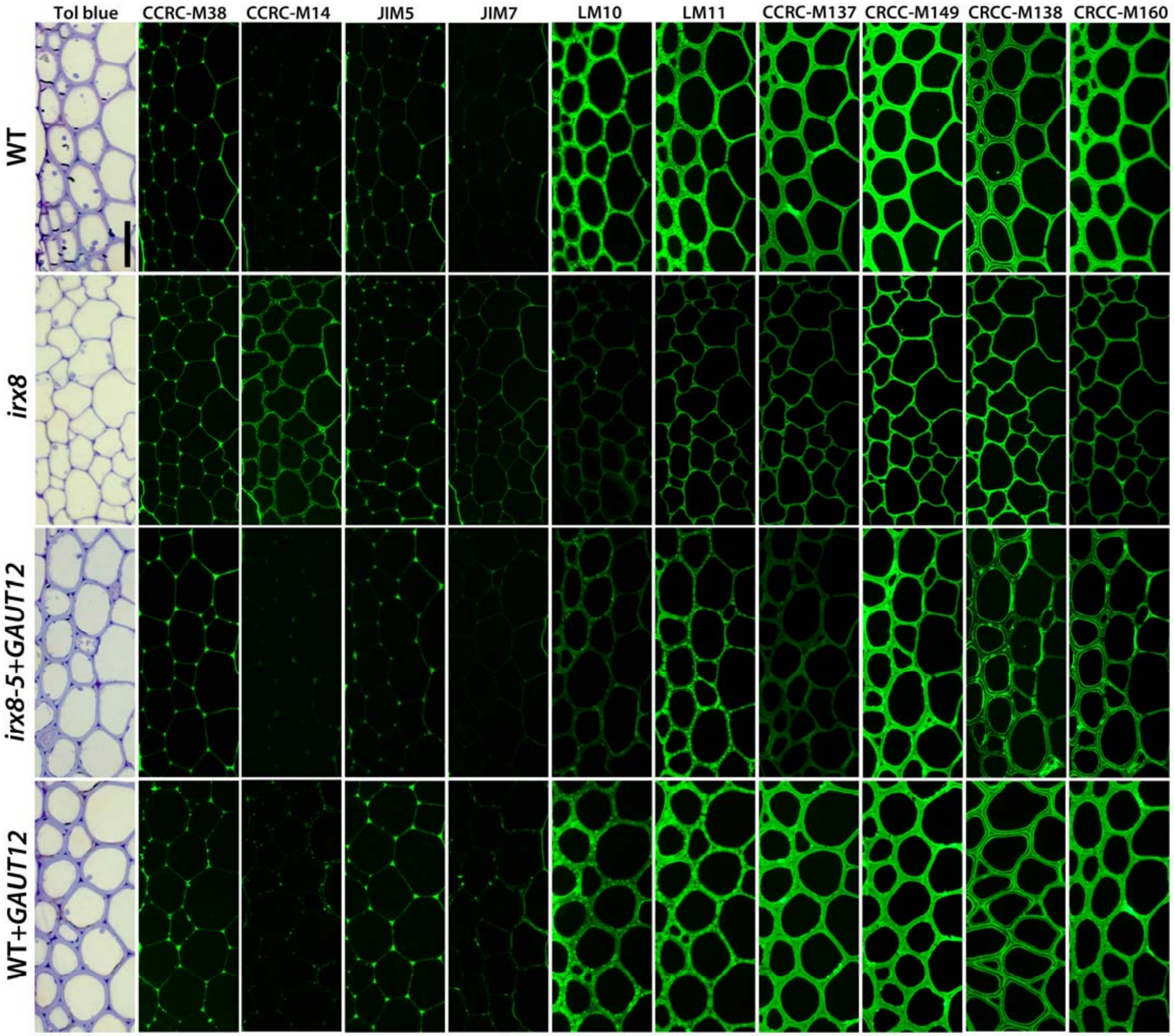
Tol blue
CCRC-M38
CCRC-M14
JIM5
JIM7
LM10
LM11
CCRC-M137
CRCC-M149
CRCC-M138
CRCC-M160
WT
irx8
irx8-5+GAUT12
WT+GAUT12

Supplemental Figure S9. Immunolabeling of interfascicular fibers from basal stems of 6-week-old wild type (WT), *irx8-5*, *irx8-5+GAUT12*, and WT+*GAUT12* plants.

CCRC-M38 recognizes un-esterified HG, CCRC-M14 recognizes RG-I backbone, JIM5 recognizes low-esterified HG, JIM7 recognizes high-esterified HG, LM10 (Xylan-6) binds to low-substituted xylan, LM11 (Xylan-6) binds to both low- and high-substituted xylan, CCRC-M149 (Xylan-7) binds strongly to xylotriose and xylopentaose, CCRC-M138 (Xylan-6) and CCRC-M160 (Xylan-7) bind to xylopentaose. The xylan epitope recognized by CCRC-M137 (xylan-7) remains undefined. Bar = 25 μm.

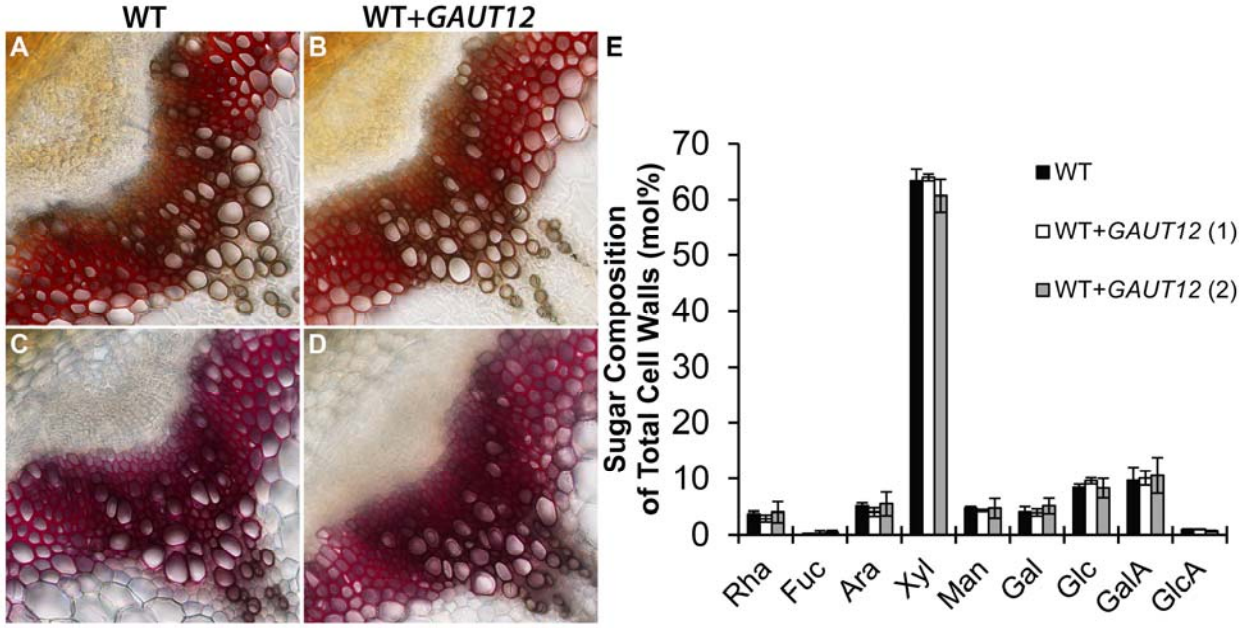

Supplemental Figure S10. Lignin staining and cell wall composition of 7-week-old WT+*GAUT12* (*GAUT12*-overexpression) plants.

WT **(A)** and WT+*GAUT12* **(B)** basal stem transverse sections stained with Mäule reagents.

WT **(C)** and WT+*GAUT12* **(D)** basal stem transverse sections stained with phloroglucinol-HCl.

(E) Sugar composition of total cell walls (AIR) of 7-week-old WT and two individual batches of WT+*GAUT12* plants represented by (1) and (2). Value = mol% of individual sugar ± SE (n=5). Neutral sugar composition was determined by gas chromatography-mass spectrometry of alditol acetates. Uronic acids were analyzed by DIONEX. Value = mol% of individual sugar ± standard error.

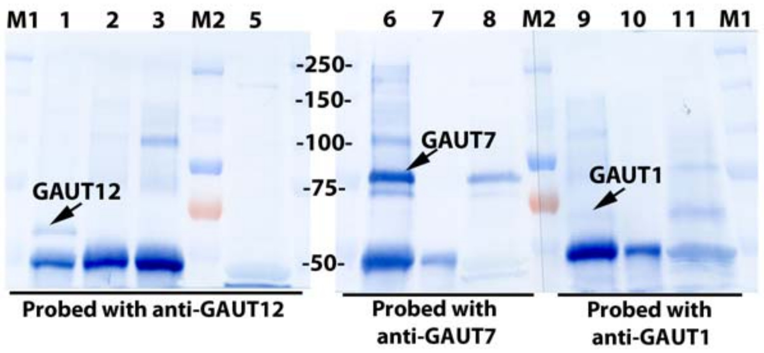

Supplemental Figure S11. Specificity of the anti-GAUT12 antibody.

Anti-GAUT12 antibody was used to immunoprecipitate (IP) GAUT12 from TX-100-treated WT Arabidopsis stem microsomal membranes. Immunoprecipitation using anti-GAUT1 and anti-GAUT7 antibodies was used as control to verify the specificity of the anti-GAUT12 antibody. The entire IP products were resolved by 10% SDS-PAGE and analyzed in Western blots. A ~58-60 kDa protein was detected in western blot analysis of the anti-GAUT12 IP material upon probing with the anti-GAUT12 antibody (lane 1). No such band was identified in the IP materials immunoabsorbed by anti-GAUT1 (lane 2) or anti-GAUT7 (lane 3) antibodies. Similarly, no GAUT1 (lane 10) or GAUT7 (lane 7) protein was detected in the anti-GAUT12 immunoabsorbed fractions. Samples are GAUT12-IP (lane 1, 7, 10), GAUT1-IP (lane 2, 9), GAUT7-IP (lane 3, 6), total WT stem microsomes (50 µg protein) (lane 5, 8, 11), and protein ladders: M1 (Bio-Rad, #161-0373) of sizes 250, 150, 100, 75, 50 kDa, and M2 (Fermentas, SM1811) of size 250, 130, 95, 72, 55 kDa.

Supplemental Table S1. Listing of primer sequences.

**Genotyping primers**	**Forward (5'-3')**	**Reverse (5'-3')**
irx8-5	GGTTTGCTTCTTGCTTCCGCT	TTTGGGACATTGACATGAATGGA
irx8-2	TTGTAACCACCAAACAGCTCC	TGAGAATCGAATGTTTTGTCG
irx9	CCAAAACTGTCAATTTATAACATTGG	ATGTTCAATGTGCCTCAAAGC
gatl1	GTTGAAGTAGCATGCTTTCCG	TATGCACAGACAAACATAGCG
Lb1	GCGTGGACCGCTTGCTGCAACT (for *gatl1*)	
Lbc	GGTGATGGTTCACGTAGTGGGCCATCGC (for *irx8-5*)	
Lb3	TAGCATCTGAATTTCATAACCAATCTCGATACAC (for *irx8-2*)	
G12 transgenics	TCTGGCATATGCTTGGTCTC	CGTTTACGTCGCCGTCCAGCTC
**Semi-qPCR primers**	**Forward (5'-3')**	**Reverse (5'-3')**
For *GAUT12* transcript analysis (**Figure S1L**)	CCATGGCACAGTTACATATATCTCCGAGCTTGAG (1)	GGTACCGCTGATGCTCTAATGTGACAGCTCTTG (2)
	CATGGGCTTATGGAATGAATG (5)	CCGCTAGGTCGAAAACATTC (3)
	TCTGGCATATGCTTGGTCTC (6)	TTGGACATGACCGTGGAAAG (4)
		CGTTTACGTCGCCGTCCAGCTC (7)
**Construct primers**	**Forward (5'-3')**	**Reverse (5'-3')**
GAUT12-EGFP	CCATGGCACAGTTACATATATCTCCGAGCTTGAG	GGTACCGCTGATGCTCTAATGTGACAGCTCTTG
**qPCR primers**	**Forward (5'-3')**	**Reverse (5'-3')**
ACTIN2	GGTAACATTGTGCTCAGTGGTGG	AACGACCTTAATCTTCATGCTGC
GAUT12	CATGGGCTTATGGAATGAATG	AGCTGCCACAAACTCAGGTC
IRX9	TACTTTGGGACCCTGAGAGATG	ACAATCTTGTGCCGGAAGTC
GATL1	CTGCAGATTGCCCCTTAATC	TCGACGTGAGCAGAAGATTG
CESA1	GGGCAGTTAAGGTGATTCCA	TTGGGTCCACATCTTCTTCC
GAUT1	CCACAAGTGGCAAAACATGA	CGCCTTGTTTAAGGGATGTG
PAL1	TATCCCGAACAGGATCAAGG	TCTCCGGTCAAAAGCTCTGT
C4H	CAGGTAGTGAAGGATTGAAATGTG	CCCACTCGATAGACCACAATG
4CL1	TTTGCTCATCGGTCATCCTG	CGACACGAATTGCTTCACATC
HCT	GCTCTTAAGGCGAAATCCAAG	CTTTCCCACTGATCTCCACAC
C3H	GTTGCTAACGTTGAAGGATCAG	TTCCGCTGTTATCGCTGTC
CCOAOMT1	CTCACAAGATCGACTTCAGGG	ACGCTTGTGGTAGTTGATGTAG
CCR1	ACCAAGTGCAAGGACGAGAA	GTCGTAGAGGCTTTGCTTGG
COMT1	TTGATCTCCCACATGTCATCG	ATGTTCGTCACTCCAGTCATG
F5H1	TCCATCAAACTTACCCGTGAC	TGTTGGACCCGTTTTAGATCC
CAD4	ATGTCTAATTATCCTATGGTTCCTGG	ACTCCGACTACATCTCCTACG
CAD5	CATCAATGGTCAACCTACACAAG	TCAACCGCCATTCCTTCTG
CAD6	TTGGGACGAAAATCGATAGC	TGCTTTTATGCCATGCTCTG

Supplemental Table S2. List of plant cell wall glycan-directed monoclonal antibodies (mAbs) used for glycome profiling analyses (Fig. 7). The groupings of antibodies are based on a hierarchical clustering of ELISA data generated from a screen of all mAbs against a panel of plant polysaccharide preparations (Pattathil et al., 2010; Pattathil et al., 2012) that groups the mAbs according to the predominant polysaccharides that they recognize. The majority of listings link to the WallMabDB plant cell wall monoclonal antibody database (http://www.wallmabdb.net) that provides detailed descriptions of each mAb, including immunogen, antibody isotype, epitope structure (to the extent known), supplier information, and related literature citations.

**Glycan Group Recognized**	**mAb Names**
Non-Fucosylated Xyloglucan-1	CCRC-M95
	CCRC-M101
Non-Fucosylated Xyloglucan-2	CCRC-M104
	CCRC-M89
	CCRC-M93
	CCRC-M87
	CCRC-M88
Non-Fucosylated Xyloglucan-3	CCRC-M100
	CCRC-M103

Non-Fucosylated Xyloglucan-4

- CCRC-M58
- CCRC-M86
- CCRC-M55
- CCRC-M52
- CCRC-M99

Non-Fucosylated Xyloglucan-5

- CCRC-M54
- CCRC-M48
- CCRC-M49
- CCRC-M96
- CCRC-M50
- CCRC-M51
- CCRC-M53

Non-Fucosylated Xyloglucan-6

- CCRC-M57

Fucosylated Xyloglucan

- CCRC-M102
- CCRC-M39
- CCRC-M106
- CCRC-M84
- CCRC-M1

Xylan-1/XG	CCRC-M111<br>CCRC-M108<br>CCRC-M109
Xylan-2	CCRC-M119<br>CCRC-M115<br>CCRC-M110<br>CCRC-M105
Xylan-3	CCRC-M117<br>CCRC-M113<br>CCRC-M120<br>CCRC-M118<br>CCRC-M116<br>CCRC-M114
Xylan-4	CCRC-M154<br>CCRC-M150
Xylan-5	CCRC-M144<br>CCRC-M146<br>CCRC-M145<br>CCRC-M155

Xylan-6	CCRC-M153<br>CCRC-M151<br>CCRC-M148<br>CCRC-M140<br>CCRC-M139<br>CCRC-M138
Xylan-7	CCRC-M160<br>CCRC-M137<br>CCRC-M152<br>CCRC-M149
Galactomannan-1	CCRC-M75<br>CCRC-M70<br>CCRC-M74
Galactomannan-2	CCRC-M166<br>CCRC-M168<br>CCRC-M174<br>CCRC-M175
Acetylated Mannan	CCRC-M169<br>CCRC-M170

β-Glucan	LAMP<br>BG1
HG Backbone-1	CCRC-M131<br>CCRC-M38<br>JIM5
HG Backbone-2	JIM136<br>JIM7
RG-I Backbone	CCRC-M69<br>CCRC-M35<br>CCRC-M36<br>CCRC-M14<br>CCRC-M129<br>CCRC-M72
Linseed Mucilage RG-I	JIM3<br>CCRC-M40<br>CCRC-M161<br>CCRC-M164

Physcomitrella Pectin	CCRC-M98<br>CCRC-M94
RG-Ia	CCRC-M5<br>CCRC-M2
RG-Ib	JIM137<br>JIM101<br>CCRC-M61<br>CCRC-M30
RG-Ic	CCRC-M23<br>CCRC-M17<br>CCRC-M19<br>CCRC-M18<br>CCRC-M56<br>CCRC-M16
RG-I/Arabinogalactan	CCRC-M60<br>CCRC-M41<br>CCRC-M80<br>CCRC-M79<br>CCRC-M44

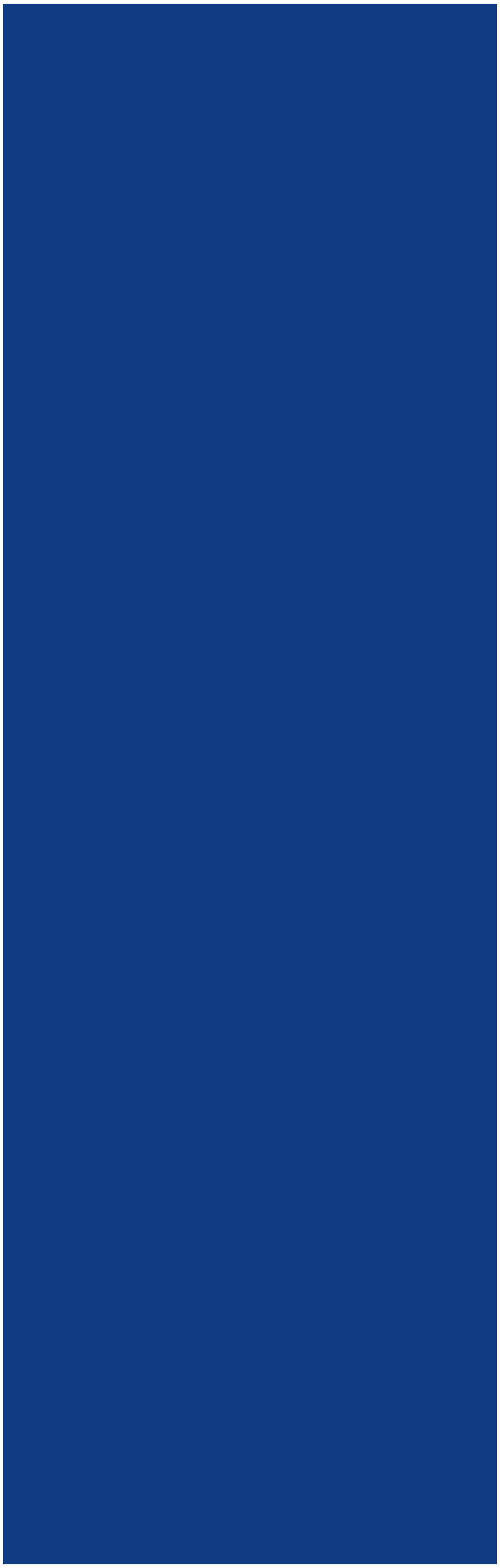

CCRC-M33

CCRC-M32

CCRC-M13

CCRC-M42

CCRC-M24

CCRC-M12

CCRC-M7

CCRC-M77

CCRC-M25

CCRC-M9

CCRC-M128

CCRC-M126

CCRC-M134

CCRC-M125

CCRC-M123

CCRC-M122

CCRC-M121

CCRC-M112

CCRC-M21

JIM131

CCRC-M22

JIM132

JIM1

CCRC-M15

CCRC-M8

JIM16

Arabinogalactan-1

JIM93

JIM94

JIM11

MAC204

JIM20

Arabinogalactan-2

JIM14

JIM19

JIM12

CCRC-M133

CCRC-M107

Arabinogalactan-3

JIM4

CCRC-M31

JIM17

CCRC-M26

JIM15

JIM8

CCRC-M85

CCRC-M81
MAC266
PN 16.4B4

Arabinogalactan-4

MAC207
JIM133
JIM13
CCRC-M92
CCRC-M91
CCRC-M78

Unidentified

MAC265
CCRC-M97

References

Pattathil S, Avci U, Baldwin D, Swennes AG, McGill JA, Popper Z, Bootten T, Albert A, Davis RH, Chennareddy C, Dong RH, O'Shea B, Rossi R, Leoff C, Freshour G, Narra R, O'Neil M, York WS, Hahn MG (2010) A comprehensive toolkit of plant cell wall glycan-directed monoclonal antibodies. Plant Physiology **153:** 514-525

Pattathil S, Avci U, Miller JS, Hahn MG (2012) Immunological approaches to plant cell wall and biomass characterization: Glycome profiling. Methods Mol Biol **908:** 61-72
